# Applications of Multi-Omics Technologies for Crop Improvement

**DOI:** 10.3389/fpls.2021.563953

**Published:** 2021-09-03

**Authors:** Yaodong Yang, Mumtaz Ali Saand, Liyun Huang, Walid Badawy Abdelaal, Jun Zhang, Yi Wu, Jing Li, Muzafar Hussain Sirohi, Fuyou Wang

**Affiliations:** ^1^Hainan Key Laboratory of Tropical Oil Crops Biology/Coconut Research Institute, Chinese Academy of Tropical Agricultural Sciences, Wenchang, China; ^2^Department of Botany, Shah Abdul Latif University, Khairpur, Pakistan

**Keywords:** multi-omics, crop sciences, genomics, transcriptomics, proteomics, ionomics, phenomics, panomics

## Abstract

Multiple “omics” approaches have emerged as successful technologies for plant systems over the last few decades. Advances in next-generation sequencing (NGS) have paved a way for a new generation of different omics, such as genomics, transcriptomics, and proteomics. However, metabolomics, ionomics, and phenomics have also been well-documented in crop science. Multi-omics approaches with high throughput techniques have played an important role in elucidating growth, senescence, yield, and the responses to biotic and abiotic stress in numerous crops. These omics approaches have been implemented in some important crops including wheat (*Triticum aestivum* L.), soybean (*Glycine max*), tomato (*Solanum lycopersicum*), barley (*Hordeum vulgare* L.), maize (*Zea mays* L.), millet (*Setaria italica* L.), cotton (*Gossypium hirsutum* L.), *Medicago truncatula*, and rice (*Oryza sativa* L.). The integration of functional genomics with other omics highlights the relationships between crop genomes and phenotypes under specific physiological and environmental conditions. The purpose of this review is to dissect the role and integration of multi-omics technologies for crop breeding science. We highlight the applications of various omics approaches, such as genomics, transcriptomics, proteomics, metabolomics, phenomics, and ionomics, and the implementation of robust methods to improve crop genetics and breeding science. Potential challenges that confront the integration of multi-omics with regard to the functional analysis of genes and their networks as well as the development of potential traits for crop improvement are discussed. The panomics platform allows for the integration of complex omics to construct models that can be used to predict complex traits. Systems biology integration with multi-omics datasets can enhance our understanding of molecular regulator networks for crop improvement. In this context, we suggest the integration of entire omics by employing the “phenotype to genotype” and “genotype to phenotype” concept. Hence, top-down (phenotype to genotype) and bottom-up (genotype to phenotype) model through integration of multi-omics with systems biology may be beneficial for crop breeding improvement under conditions of environmental stresses.

## Introduction

Various promising omics technologies have emerged over the last few decades. These omics-based approaches have proved themselves to be valuable for exploring the genetic and molecular basis of crop development through modifications in DNA, transcript levels, proteins, metabolites, and mineral nutrient against a backdrop of environmental and physiological stress responses (Muthamilarasan et al., 2019). Several omics approaches, such as genomics, mutagenomics, transcriptomics, proteomics, metabolomics, phenomics, and ionomics, have revealed each corresponding molecular biological facet integrated with plant systems (Salt et al., 2008; Houle et al., 2010; Talukdar and Sinjushin, 2015; Wu et al., 2017; Muthamilarasan et al., 2019). The advent of next-generation sequencing (NGS) technologies has led to high throughput and rapid data generation for genomes, epigenomes, transcriptomes, proteomes, metabolomes, and phenomes (Großkinsky et al., 2018). The integration of multiple omics approaches could elucidate gene functions and networks under conditions of physiological and environmental stress (Singh et al., 2013). Comprehensive multi-omics approaches with robust techniques have been used to identify and decipher essential components of stress responses, senescence, and yields in various economically important crops including wheat, soybean, and millet (Deshmukh et al., 2014; Talukdar and Sinjushin, 2015; Muthamilarasan and Prasad, 2017; Shah et al., 2018; Yadav et al., 2018).

**Figure 1:**
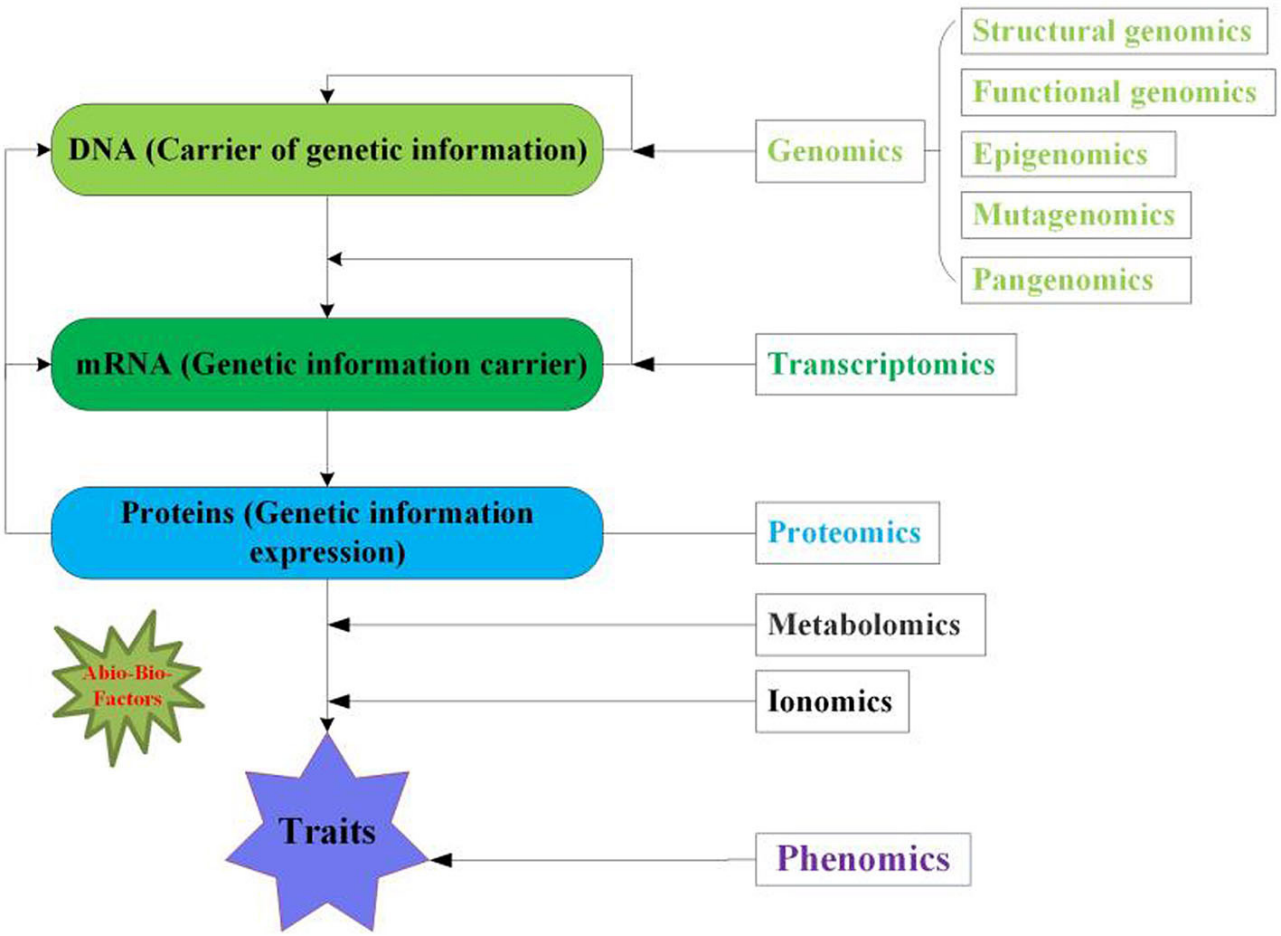
Overview of multi-omics approaches for crops. Genomics reflects DNA the genetic information contains five aspects including structural, functional, epigenomics, mutagenomics and pangenomics. Transcriptomics denotes the mRNA (transcript) the carrier of genetic information for translation. Proteomics symbolizes the protein the expression of genetic information. While, metabolomics and ionomics are linkage between proteomics and phenomics. Phenomics displays the phenotype of crop traits. The green asterisk indicates the abiotic and biotic factors influence the multi-omics.

**Figure 2:**
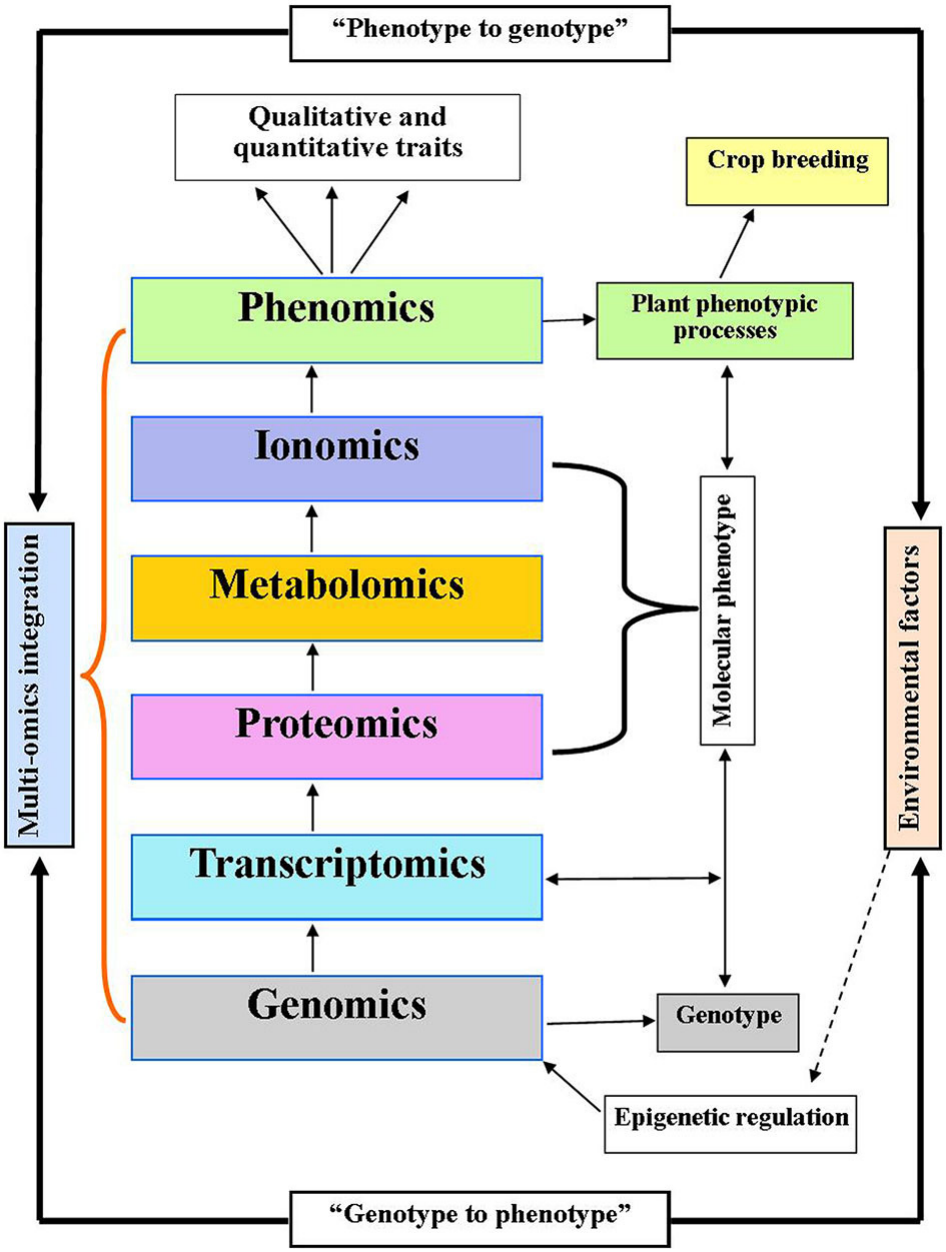
Integration of multi-omics model linking genotype to phenotype and phenotype to genotype concept with systems biology. Genomics indicates the genotype which determines phenotype traits (crop breeding improvement) via transcriptome, proteome, and metabolome. While, proteome, metabolome, and ionome are supposed to be a molecular phenotype. Environmental factors may trigger the regulatory events through epigenetic regulation of genome.

In this review, we discuss multi-omics approaches, their applications, and anticipated implementations in crop science to improve crop yields and enhanced biotic and abiotic stress tolerance (Figure 1). We propose that the integration of entire omics approaches could provide a basis to improve genetic development, crop yields, crop breeding science, and crop resistance to physiological and environmental stress (Figure 2).

## Omics Technologies for Crop Improvement

### Genomics

Genomics deals with the study of genes and genomes and focuses on the structure, function, evolution, mapping, epigenomic, mutagenomic, and genome editing aspects (Muthamilarasan et al., 2019). Genomics can play an indispensable role in elucidating genetic variation, which may enhance crop breeding efficiency and subsequently result in the genetic improvement of crop species. Structural genomics encompasses sequence polymorphism and chromosomal organization and enables the construction of physical and genetic maps to identify traits of interest for plant biologists. In contrast, functional genomics provides insights into the functions of genes with regard to the regulation of the trait of interest. When epigenetic changes occur in the form of histone modifications, DNA, or small RNA methylations at the genomic level, the phenomenon is known as epigenomics. Mutagenomics deals with mutational events orchestrating genetic modification in mutant traits. However, pangenomics defined as sum of a core genome, shared by all individuals, plus a dispensable genome partially shared or individual specific (Tettelin et al., 2005). Mutagenomics and pangenomics have emerged as recent omics approaches focused on mutagenesis and the pangenome in crop sciences, respectively (Golicz et al., 2016a; Goh, 2018; Muthamilarasan et al., 2019).

### Structural Genomics

Structural genomics depends on molecular markers that may be useful for tagging and mapping genes of interest and their subsequent deployment in crop breeding programs. The marker techniques can be categorized into classes. The first one is non-PCR-based techniques which include restriction fragment length polymorphisms (RFLP). Restriction fragment length polymorphism detects DNA polymorphism through hybridizing labeled DNA probe to a Southern blot of DNA digested by restriction enzymes and resulting in differential DNA fragment profile (Agarwal et al., 2008). The second one is PCR-based techniques for markers such as, random amplified polymorphic DNA (RAPD), amplified fragment length polymorphisms (AFLP), and single nucleotide polymorphisms (SNPs) (Williams et al., 1990; Vos et al., 1995). The RAPD markers are PCR-based amplification of random DNA segments using single primer of arbitrary nucleotide sequence (Rabouam et al., 1999). Amplified fragment length polymorphisms is also the PCR-based technique which conducts selective PCR amplification of restriction fragments from a total digest of genomic DNA (Vos et al., 1995). Single nucleotide polymorphisms defined as single nucleotide variations in genome of an individual or an organism. The SNP may be performed through sequencing of genomic PCR products derived from varied individuals (Appleby et al., 2009). Whereas, the diversity arrays technology (DArT) a high-throughput technique which is based on microarray hybridization involving genotyping of numerous polymorphic loci spread over the genome (Jaccoud et al., 2001). The identification and usage of SNPs became possible with the advent of NGS.

Approaches utilized to understand and study the multiple traits in crops are quantitative trait loci (QTL) mapping and genome-wide association studies (GWAS). Quantitative trait loci mapping is a statistical method which assists in linking two types of data, i.e., complex phenotypes with genotypes. Molecular markers such as SNPs and AFLPs are commonly utilized for mapping QTLs, and then these may be correlated with observed phenotypic data (Kearsey, 1998; Challa and Neelapu, 2018). However, GWAS could identify variants associated with traits. Genome-wide association studies may also identify correlation between the genetic variants/phenotypes in a population of any organism based on SNPs in the sequence data (Challa and Neelapu, 2018).

The role of GWAS in genomics approaches is indispensable for enhancing the tolerance of crops to abiotic stress [e.g., the use of GWAS to evaluate how multiple abiotic stressors affect the oil content in sunflowers (*Helianthus annuus* L.) (Mangin et al., 2017)]. Previously, GWAS identified 48 QTLs related to the yield of maize crop under heat and water stress (Millet et al., 2016). Genome wide associations with environmental variables were used to predict the SNPs in sorghum (*Sorghum bicolor*) that were associated with drought stress (Lasky et al., 2015). Another GWAS identified 213 unique genomic regions associated with drought tolerance in sorghum (Spindel et al., 2018). Genome-wide association studies have also identified the (drought resistance) DR-related loci in rice crop (Guo et al., 2018). Moreover, numerous SNPs associated with drought-responsive TFs have been identified using GWAS of maize crop (Shikha et al., 2017). In addition, structural variants (SVs) play a vital role in the genetic control of agronomically essential traits in crops. The association of SVs with agronomical traits has been reported in GWAS of *B. napus* (Gabur et al., 2018), maize (Lu et al., 2015), and soybean (Zhou et al., 2015).

Breeders are now capable of enhancing hybrid breeding through marker-assisted selection (MAS) with genotyping-by-sequencing (GBS) to increase crop quality and yield (He et al., 2014). Multiparent mapping, in particular multiparent advanced generation intercrosses (MAGIC) and nested association mapping (NAM) in model plants and crops (Yu et al., 2008; Kover et al., 2009), has been able to expose the large amount of phenotypic diversity that may be achieved through experimental studies. The MAGIC population is ideal for breeding improvement. Analyses of the relationships between genotypes and phenotypes are able to identify QTLs that may be subsequently authenticated utilizing functional genomics approaches.

### Functional Genomics and Mutagenomics

The vast resources and information provided through structural genomics will ultimately be utilized by functional genomics. Functional genomics refers to development of global experimental approaches to assess the function of gene (Hieter and Boguski, 1997). Numerous biotechnological tools have been developed to identify and isolate genes of interest, to clone and characterize those genes, and to overexpress or knock-out lines for functional transgenic analyses (Muthamilarasan et al., 2019). Prior to genome sequencing approaches, the identification of candidate genes involved arduous procedures including suppression subtractive hybridization (SSH), expressed sequence tag (EST), and cDNA-AFLP-sequencing. Subsequently, the tediousness of these approaches decreased with the introduction of NGS (Muthamilarasan et al., 2019).

The access to crop genome sequencing has identified genes that play their role in disease resistance, stress resistance, and yield determination. Furthermore, authentic genome engineering has been envisaged to improve crops by utilizing genome editing tools such as the clustered regularly interspaced short palindromic repeats (CRISPR/Cas9 system) and transcription activator-like effector nuclease (TALEN) (Rinaldo and Ayliffe, 2015). Genome editing tools without the insertions of foreign DNA could possibly enhance yield via the introduction of pest and disease resistance in genetically modified crops. Using TALEN and CRISPR/Cas9 technologies, a bread wheat mildew resistance locus o (TaMlo) mutant was generated (Wang et al., 2014). Similarly, the same technique was adopted with tomato crop to create an SlMlo mutant (Nekrasov et al., 2017). Genome editing with the CRISPR/Cas9 system has already been reported for numerous important crops including soybean, rice, maize, and sorghum (Jiang et al., 2013; Lawrenson et al., 2015; Li et al., 2015; Svitashev et al., 2015). Virus induced gene silencing (VIGS) is a reverse genetic technique to analyze the functions of genes that manifest in tomato crop in response to biotic and abiotic stress (Saand et al., 2015). Through comparative genomics, various mutants have been identified that are related to crop growth, development, and stress tolerance in rice, maize, wheat, and barley (Talukdar and Sinjushin, 2015). A soybean mutation has been found to alter the transcriptomic profiling of GmNARK (*Glycine max* leucine-rich repeat receptor kinase) rhizobia independent nodulation through the jasmonate pathway (Pathan and Sleper, 2008).

Mutagenomics emerged as a modern omics approach which enables to study mutational events orchestrating genetic modification in mutant traits. Such mutational events may be characterized by using high-throughput genomics technologies including serial analysis of gene expression (SAGE), high resolution melt (HRM), Targeted Induced Local Lesions IN Genomes (TILLING), and microarray analysis (Penna and Jain, 2017). Targeted Induced Local Lesions IN Genomes (McCallum et al., 2000) in functional genomics has characterized mutagenesis and offers high throughput mutations in crops (Henikoff et al., 2004; Mba, 2013). Initially, TILLING technology was developed as a functional genomics strategy, but soon, it became a useful tool for crop breeding as an alternative to the transgenic approaches (Kurowska et al., 2011). The feasibility of using this technique has been documented in numerous essential crops, such as soybean wheat, rice, tomato, rapeseed (*Brassica napus*), and sunflower (Kurowska et al., 2011; Witzel et al., 2015). Thus, this technique has proved to be a potential method for functional genetics as well as a valuable tool to improve crop breeding (Chen et al., 2014). Mutants controlling the seed oil composition were screened through the reverse genetics technique TILLING (Knoll et al., 2011; Kumar et al., 2013). For example, mutants rich in oils, isoflavones, and oleic acids (FAD2 and FAD2-1B) have also been isolated in soybean crop (Pathan and Sleper, 2008). TILLING has also been applied to detect mutations in numerous crops including rice (Suzuki et al., 2008), maize (Till et al., 2004), wheat (Dong et al., 2009), barley (Caldwell et al., 2004), tomato (Minoia et al., 2010), and soybean (Cooper et al., 2008).

Several microarray analyses showed that plant mutagenesis could induce more transcriptomic changes compared with those due to transgene insertion (Varshney et al., 2010). Mutagenesis constitutes a vital technique to identify gene functions and develop countless agronomic traits with desirable variations (Henikoff et al., 2004; Varshney et al., 2010). Approximately 3,000 mutant varieties of various crops have been developed globally, of which 776 mutants ensure nutritional quality (Jain and Suprasanna, 2011). With improvements in functional, biological, and breeding tools, the mutagenomics has ensured induced mutagenesis in crops. However, various mutant traits have been identified in crop plants in perspective of global impact of mutation-derived varieties on food production and quality enhancement (Ahloowalia et al., 2004).

Mutagenomics through reverse genetic approaches have provided opportunity to silence and interrupt the candidate genes to investigate the function of gene. The specific reverse genetic techniques utilized to screen/induce mutations for crops that include, RNA Interference (RNAi) and (VIGS). When mutant alleles are not available, the reverse genetic techniques can be used to knockdown or silence the phenotype of gene, allowing analysis of gene function (Talukdar and Sinjushin, 2015). Furthermore, those reverse genetic approaches have been utilized to screen for mutations in wheat, rice, maize, barley, tomato, sunflower, cotton, chickpea (*Cicer arietinum* L.), pea (*Pisum sativum* L.), and soybean crops including RNAi and gene silencing technologies (Dwivedi et al., 2008; Gupta et al., 2008; Tomlekova, 2010).

As such, both functional genomics and mutagenomics have been found to be useful for improving crop growth, yield, and stress resistance.

### Epigenomics

The epigenetics refers to heritable changes other than those in the DNA sequence. These epigenetic changes brought about through DNA methylation and post-translational modification (PTM) of histones (Strahl and Allis, 2000; Novik et al., 2002). The merger of epigenetics and genomics is known as epigenomics which has arisen as new omics technique in order to understand the genetic regulation and its contribution to cellular growth and stress responses (Callinan and Feinberg, 2006). Unlike genomics, epigenomics may be influenced by environmental factors, including abiotic and biotic stress. Nevertheless, genome level studies could be conducted to analyze these epigenetic events at any developmental stage or to evaluate abnormalities due to plant disease (Muthamilarasan et al., 2019). The bisulfite sequencing technique can identify the DNA methylation status of the genome (Cokus et al., 2014) and has been successfully validated in tomato, maize, and soybean crops with regard to DNA methylation and chromatin regulated genes (Gent et al., 2013; González et al., 2013; Schmitz et al., 2013). The quantification of DNA methylation in the genome through a methylation-sensitive amplified polymorphism (MSAP) approach is common and has been performed in wheat and foxtail millet crops under salinity stress (Zhong et al., 2009; Pandey et al., 2017). Moreover, chromatin immunoprecipitation sequencing (ChIP-Seq) is a unique approach for the analysis of histone proteins and DNA methylation (van Dijk et al., 2010) and has been used in rice crop under drought stress (Zong et al., 2013). DNA methylation studies have been carried out with epigenome modifications associated with ripening in tomato and tissue cultured stable epigenome changes in rice crop (Stroud et al., 2013; Zhong et al., 2013). One epigenomic study found this approach to be beneficial for identifying histone modifications associated with photosynthesis in maize (Offermann et al., 2006).

Recently, an epigenome wide association study identified the MANTLED locus responsible for the mantled phenotype (hypomethylation) in the oil palm (*Elaeis guineensis*) (Ong-Abdullah et al., 2015). Karma (LINE) retrotransposon methylation was linked with normal fruit yield clones compared to mantled clones (Ong-Abdullah et al., 2015). Whole-genome bisulfite sequencing (WGBS) identified ncRNAs in cotton crop under drought stress (Lu et al., 2017). Taken together these data indicate that applications of epigenomics could play important role in crops improvement in response to environmental stresses.

### Pangenomics

The pangenome concept refers to the full genomic makeup of a species, which can be divided into a set of core and dispensable genes. The sets of core genes are shared by all individuals, whereas, set of dispensable genes (also known as accessory genes) are individual-specific and/or present in some individuals but not all (Tettelin et al., 2005). Advancements in sequencing technology and analysis tools have made it possible to sequence several accessions of crop species (Golicz et al., 2016a). A wave of pangenomic studies in crops including rice (Schatz et al., 2014; Wang et al., 2018; Zhao et al., 2018), soybean (Li et al., 2014), wheat (Montenegro et al., 2017), maize (Hirsch et al., 2014), *Brassica napus* (Hurgobin et al., 2018), and *Brassica rapa* (Lin et al., 2014) have revealed that dispensable genes play important roles in maintaining crop diversity and improving quality. A pangenomic study using nine morphologically diverse *Brassica oleracea* varieties and a wild relative demonstrated that several variable genes were annotated with functions related to major agronomic traits, such as glucosinolate metabolism, vitamin biosynthesis, and disease resistance (Golicz et al., 2016b). Numerous methodologies have been used to generate pangenomes in crops and their wild relatives, such as comparative *de novo*, iterative assembly and “map-to-pan.” Comparative *de novo* approaches have been conducted with soybean and rice and their wild relative in order to analyze the genetic variation and agronomic traits (Li et al., 2014; Zhao et al., 2018). While an iterative assembly approach was performed with *B. oleracea* (Golicz et al., 2016b), bread wheat (Montenegro et al., 2017), and *B. napus* (Hurgobin et al., 2018), and a “map-to-pan” approach was used with rice (Wang et al., 2018).

Pangenomic studies have recently been investigated to evaluate the genetic diversity of crop species. Comparatively dispensable genes tend to be more variable than core genes. For example, higher densities of SNPs and insertions/deletions (InDels) have been found in sets of dispensable genes when compared to those in sets of core genes in *Brachypodium distachyon* (Gordon et al., 2017), rice (Wang et al., 2018), and soybean (Li et al., 2014). The dispensable genes of a pangenome are determined by structural variation (Xu et al., 2012; Mace et al., 2013), and dispensable genomes have been found to be enriched with genes related to disease resistance in crops such as maize (Zuo et al., 2015) and rice (Fukuoka et al., 2009) and abiotic stress in barley (Francia et al., 2016) and sorghum (Magalhaes et al., 2007). Furthermore, pangenomics may be used to improve crops. Multiple crop wild relatives (CWRs) have been used in breeding programs specifically for their quantitative and adaptive traits. Traits associated with yield and its components (e.g., grain size) were subject to intensive selection during domestication and breeding improvement in crops including rice (Xiao et al., 1998), sorghum (Tao et al., 2017, 2018), and wheat (Huang et al., 2003). Several wild relatives have also been found to be able to contribute genes to improve traits, such as grain quality (Campbell et al., 2016) and biotic/abiotic stress resistance in crops (Zhang et al., 2006; Ram et al., 2007; Cao et al., 2011; Huang et al., 2013). Thus, pangenomic studies could be used to mine elite genes in CWRs for crop improvement.

### Transcriptomics

Transcriptomics deals with transcriptome that refers to the complete set of RNA transcripts which are produced by genome of an organism in a cell or tissue (Raza et al., 2021). Transcriptome profiling is dynamic and has emerged as a promising technique to analyze gene expression in response to any stimuli over a certain time period (Duque et al., 2013; El-Metwally et al., 2014). This strategy helps the researcher to observe the differential expression of genes *in vitro* to understand the first layer function of a particular gene. Initially, transcriptome dynamics were analyzed using traditional profiling, cDNAs-AFLP, differential display-PCR (DD-PCR), and SSH, but these techniques provided low resolution (Nataraja et al., 2017). Soon after, the introduction of robust techniques made it possible for RNA expression profiling utilizing microarrays, digital gene expression profiling, NGS, RNAseq, and SAGE (Kawahara et al., 2012; De Cremer et al., 2013; Duque et al., 2013).

Microarray analysis has revealed the differentially expression of genes in soybean and barley during developmental and reproductive stages, respectively, under drought stress (Guo et al., 2009; Le et al., 2012). Similarly, the differential expression of genes was identified in soybean under dehydration stress using an Affymetrix GeneChip array (Khan et al., 2017). The expression of genes has been found to alter various TFs in Arabidopsis, soybean, and rice crops in response to abiotic stress (Xiong et al., 2002; Wohlbach et al., 2008). The novel TFs, Cys-2/His-2-type zinc finger (C2H2-ZF) TF and drought and salt tolerance (DST), were found to control stomatal aperture in response to salt and drought stress in rice crop (Huang et al., 2009). Another, study demonstrated the function of WRKY TFs in response to abiotic stress in wheat (Okay et al., 2014). Although phytohormones, non-coding RNAs, and small peptides regulate the expression of genes and are considered to be key components that execute gene functions in response to abiotic stress conditions in *Arabidopsis* and model crops including rice, tomato and wheat (Matsui et al., 2008; Chekanova, 2015; Bashir et al., 2019). Various phytohormone-independent abiotic stress responses are regulated by several TFs, such as DRE-/CRT-binding protein 2 (DREB2) and dehydration-responsive element/C-repeat (DRE/CRT), in rice crop (Todaka et al., 2015).

Transcriptome studies in sorghum revealed a set of differentially expressed (DE) genes in response to drought, heat, and osmotic stress as well as hormone treatment (Dugas et al., 2011; Johnson et al., 2014). Similarly, differential expression patterns of OsMADS genes were found in developing rice crop in response to drought stress (Jin et al., 2013). These transcriptome sequencing analyses have shown differential expression during growth and in response to stress and may be useful for functional analyses. Therefore, these reports demonstrate the role of transcriptomics in terms of stress responses and development for crops.

Novel advancements in transcriptomics have been brought about through *in situ* RNA-seq (i.e., *in situ* ligation), in which RNA is sequenced in living cells or tissues (Ke et al., 2013). Spatially resolved transcriptomics is another technique that detects gene expression with spatial information within cells or tissues to provide a comprehensive molecular description of physiological processes in organisms (Burgess, 2015). Numerous RNA-seq analyses have unveiled tissue-specific expression in response to abiotic and biotic stress in foxtail millet and sweet potato (*Impomoea batatas* L.) crops (Qi et al., 2013; Hittalmani et al., 2014; Bonthala et al., 2016; Li et al., 2017). Total RNAseq has shown DE genes and SSR markers during the development of cowpea (*Vigna unguiculata* L. Walp.) crop (Chen et al., 2017). Thus, RNA-seq has proved to be one of the better techniques of transcriptomics to develop genic-SSR markers that can be linked to phenotypic traits connected with the candidate genes. Moreover, various studies utilizing RNA-seq technique have been conducted in rice, maize, and rapeseed oil to identify drought stress responsive genes (Kakumanu et al., 2012; Huang et al., 2014; Bhardwaj et al., 2015).

Comparative transcriptomics is another means to understand differential expression profiles in response to stress in different crop species. Comparative transcriptomic analysis has identified sixteen common genes in rice, wheat, and maize compared with those in switch grass in response to heat stress (Ding et al., 2013; Li et al., 2013). Comparative transcriptome and microarray analyses of biotic and abiotic stress and hormonal treatments have revealed multiple cross-talk pathways in cotton and potato (*Solanum tuberosum* L.) crops (Massa et al., 2013; Zhu et al., 2013). As such, these regulatory networks among stress tolerance genes might be beneficial for improving crops.

Recently, an alternative splicing (AS) transcriptomics approach was launched to generate multiple transcripts in response to abiotic stress conditions (Laloum et al., 2018). This method has been applied in crops including rice, maize, and sorghum in response to heat and drought stress (Zhang et al., 2015). Hence, AS transcriptomic analyses demonstrated the role for splicing factors controlling abiotic stress responses in crops. Collectively, these all transcriptomic techniques could play a vital role in the regulation of gene expression leading to the improvement of crop species.

### Proteomics

Proteomics is a technique involved in the profiling of total expressed protein in an organism and is divided into four different parts including sequence, structural, functional, and expression proteomics (Mosa et al., 2017; Aizat and Hassan, 2018). Sequence proteomics determines the amino acid sequences that are usually identified sequentially utilizing high performance liquid chromatography (HPLC; Twyman, 2013). Structural proteomics deals with the structure of proteins to understand their putative functions. Structural proteomics can be analyzed through several approaches, such as computer based modeling, and experimental methods including nuclear magnetic resonance (NMR), crystallization, electron microscopy, and the X-ray diffraction of protein crystals (Sali et al., 2003; Woolfson, 2018). Functional proteomics determines the functions of a protein, and those functions are examined through various methods, such as yeast-one or two hybrids and protein microarray profiling (Lueong et al., 2014).

Advancements in protein extraction and separation have contributed to the rapid improvements of plant proteomic research, at both sample and genome-wide scales (Nakagami et al., 2012). Conventional proteomics are chromatography based techniques; which include exchange chromatography (IEC), size exclusion chromatography (SEC), and affinity chromatography. However, western blotting and enzyme-linked immunosorbent assay (ELISA) could be used for selective proteins analysis. Later, some more advanced techniques such as SDS-PAGE, two-dimensional gel electrophoresis (2-DE), and two-dimensional differential gel electrophoresis (2D-DIGE) were developed and used through gel based techniques for separation of proteins. Simultaneously, for rapid protein expression analysis the protein microarrays/chips have been devised for detection of small amount of protein sample. Moreover, stable isotope labeling with amino acids in cell culture (SILAC), Isotope-coded affinity tag (ICAT) labeling and isobaric tag for relative and absolute quantitation (iTRAQ) have been developed as advanced techniques for quantitative proteomic analysis. Recently, two major high-throughput approaches including X-ray crystallography and NMR spectroscopy have been developed for three-dimensional structure determination of proteins that may be useful to understand the biological function of proteins (Aslam et al., 2017). Applications and importance from conventional to modern proteomic approaches have been discussed below.

Two-dimensional gel electrophoresis and SDS-PAGE are required to identify the proteins and measure the quantitative parameters of protein content, respectively (Eldakak et al., 2013). Henceforth, the identified proteins are used to analyze the molecular mass of peptides with mass-spectrometry (MS), ion trap-mass spectrometry (IT-MS), or liquid-chromatography (LC; Fournier et al., 2007). Additionally, the molecular weights of proteins have been identified using MALDI-TOF, electrospray ionization (ESI), and collision-induced dissociation (CID; Tanaka et al., 1988; McLuckey and Stephenson, 1998; Baggerman et al., 2005).

Functional proteomics approaches have identified ROS scavengers including quinone redcutase, γ-glutamylcysteine synthetase, dehydrins, and dehydroascorbate reductase in tomato and sunflower crops (Shalata et al., 2001; Di-Baccio et al., 2004; Mittova et al., 2004). Meanwhile, molecular chaperones, such as heat shock proteins, have also been identified during proteome analyses in wheat and sugarcane (Demirevska et al., 2008; Jangpromma et al., 2010). Various drought sensitive and tolerant rice cultivars have been identified through comprehensive proteomics studies (Salekdeh et al., 2002; Rabello et al., 2008; Muthurajan et al., 2011; Maksup et al., 2014). Therefore, functional proteomics studies depict their significant role in crop defense response.

In quantitative proteomics, the iTRAQ method has demonstrated the differential expression of proteins in potato crop under abiotic stress (Liu et al., 2015). Recently, an iTRAQ-based comparative proteomic analysis of two coconut varieties identified numerous stress-responsive DEPs in two varieties of coconuts (Yang et al., 2020). In addition, iTRAQ-based proteomic analysis has provided new insights into somatic embryogenesis in cotton crop (Zhu et al., 2018). Thus, iTRAQ-based quantitative proteomic studies also play important role for crops against abiotic stresses.

A proteomics approach in response to the presence of pathogens was established with Vitis species (Basha et al., 2010). A post-iTRAQ-based comparative proteomic analysis was used to identify translational modifications (e.g., phosphorylation and ubiquitination) and protein–protein interactions that occur in biological or molecular mechanisms within cells. Phosphoproteomics intends to analyze protein phosphorylation through detecting phosphoproteins and their phosphorylated amino acid residues in a quantitative or qualitative manner (Mosa et al., 2017). In addition, phosphoproteomics has also been associated with protein functions, and thus it may play a role in the identification of pathways involved in various cell functions (Mosa et al., 2017). Phosphoproteomics, along with proteins have revealed large numbers of drought stress-related proteins in two wheat crop cultivars (Zhang et al., 2014a). Further, proteomics and phosphoproteomics have been combined to investigate diverse functions in crops [e.g., wheat and grapevine (*Vitis vinifera* L.)] in response to phytoplasma and (*Septoria tritici*) fungal pathogen (Margaria et al., 2013; Yang et al., 2013). The wheat varieties against both drought and phytoplasma stresses showed resistant (Yang et al., 2013; Zhang et al., 2014a), whereas, grapevine was susceptible to phytoplasma infection (Margaria et al., 2013). Thus, phosphoproteomics could be helpful in order to identify resistant and/or susceptible crop cultivars against various stresses. Moreover, a combined proteomics and metabolomics approach with functional genomics in legumes has provided an understanding of the stress biology of these crops and the identification of molecular markers for legume smart breeding programs (Ramalingam et al., 2015). Hence, proteomics plays a vital role in deciphering functional mechanisms in crop science against diverse stresses and can help to improve crop yields.

Using LC-MS/MS, one proteomic study identified 75 differentially expressed proteins (DEPs) in a dehydration-sensitive chickpea cultivar (Subba et al., 2013). The majority of DEPs were involved in molecular chaperons, cell signaling, gene transcription, and regulation and ROS catabolic enzymes (Subba et al., 2013). Comparative proteomics and gene expression analyses using 2-DE along with LC-MS/MS have also identified DEPs associated with abiotic stress responses in chickpea (Arefian et al., 2019). By applying non-gel-based LC-MS/MS approaches, a large number of nodule proteins were identified in response to drought stress in *Medicago* spp. (Larrainzar et al., 2007). Later, the same method was used for the relative quantification of root nodule proteins in *Medicago* spp. (Larrainzar et al., 2009). Furthermore, using 2D-GE and ESI-LC-MS/MS approaches, numerous DEPs were identified in response to abiotic stress in legume crops, such as chickpea, common bean, and *M. truncatula* (Ramalingam et al., 2015). Several proteomics approaches, such as MALDI-TOF, SDS-PAGE, MS, 2-DE, and PMF have been applied in rapeseed, soybean, wheat, sugarcane, and cotton to determine stress-response pathways (Demirevska et al., 2008; Toorchi et al., 2009; Jangpromma et al., 2010; Nouri and Komatsu, 2010; Deeba et al., 2012; Mohammadi et al., 2012). These proteomics techniques have been implemented in plants under drought stress (Ghosh and Xu, 2014). However, 2-DE and SDS-PAGE proteomics techniques have been implemented in plants under drought stress (Ghosh and Xu, 2014). Importantly, numerous techniques (i.e., 2D-GE, MALDI-TOF, SDS-PAGE, ESI-IT- LC-MS/MS, and iTRAQ) used for cereal crops such as barley, maize, pearl millet, rice, sorghum, and wheat under drought stress response have been reviewed comprehensively by Ghatak et al. (2017). Hence, such proteomic studies revealed their role for crops in response to diverse abiotic stress conditions.

### Metabolomics

Metabolomics defined as the comprehensive study of metabolites which participate in different cellular events in a biological system. However, metabolome denotes the complete set of metabolites synthesized via metabolic pathways in plant system (Fiehn, 2002; Baharum and Azizan, 2018). Next-generation sequencing technologies have emerged as promising tools in order to understand the regulation of gene expression and molecular basis of cellular responses which occur in crops in response to biotic and abiotic stresses (Abdelrahman et al., 2018). However, metabolomics in combination with NGS has provided a basis to predict an initial metabolic network from a genome sequence of an organism (Weckwerth, 2011b). The genome sequencing approach (NGS) and quantification of metabolites (through MS) integrated the information in order to develop strategies for crop-improvement (Pandey et al., 2016).

Metabolites may be viewed as the end products of gene expression that display the biochemical phenotype of the cell (Weckwerth and Fiehn, 2002). Proteomics recognizes only gene products, whereas metabolomics may determine the expression of proteins metabolically and identify the biochemical processes that play important roles in gene functioning (Weckwerth and Morgenthal, 2005; Lindon and Nicholson, 2008).

Metabolites possess different chemical and physical properties; hence, separation and analytical techniques are required to generate metabolic profiles of a given plant sample (Jogaiah et al., 2013). Several analytical techniques have been implemented in plant systems to quantify metabolites including thin layer chromatography (TLC), gas/liquid-chromatography-mass spectrometry (GC/LC-MS), liquid chromatography-electrochemistry-mass spectrometry (LC-EC-MS), NMR, direct infusion mass spectrometry (DIMS), Fourier-transfer infrared (FT-IR), and capillary electrophoresis-liquid- chromatography mass spectrometry (CE-MS; Fiehn et al., 2000; Weckwerth, 2003; Moco et al., 2007; Allwood and Goodacre, 2010; Saito and Matsuda, 2010; Duque et al., 2013; Jogaiah et al., 2013). The CE-MS, GC-MS, LC-MS, and NMR techniques are the most frequently used in plant metabolomics (Fiehn, 2002; Kikuchi and Hirayama, 2007; Moco et al., 2007; Allwood and Goodacre, 2010; Weckwerth, 2010; Kim et al., 2011). These techniques depend on the selectivity, sensitivity, speed, and accuracy of the approach. NMR is fast and selective, although mass spectrometry techniques (CE-MS, GC-MS, and LC-MS) are suitably sensitive and selective but supposed to be time consuming (Sauter et al., 1991; Sumner et al., 2003).

Annotation and reporting of metabolomics data is an important in order to identify and analyze metabolites properly. However, recently, Alseekh et al. (2021) reported guidelines for annotation and quantification of LC/GC-MS-based metabolomics data reporting. Their recommended guidelines (i.e., sample preparation, sample replication and randomization, quantification, recovery and recombination, ion suppression, and peak misidentification) could be an effective tool/method for acquisition and reporting of metabolite data. Nonetheless, workflow for sampling, quenching, metabolite extraction, and storage has also been elucidated. The adopting certain recommendations may avoid misinterpretation of metabolite data and ensure the reporting transparency in LC/GC-MS-based metabolomics-derived data (Alseekh et al., 2021).

Plants have large chemically complex machinery that they employ as a major defense system against abiotic stress and pathogens. The mechanisms of plant metabolic responses to stress depend on plant–stress or pathogen interactions. The pivotal role of metabolites in cereal crops, such as rice, maize, and barley, has been identified in the presence of various biotic stressors (Balmerl et al., 2013). The metabolic profiles of three rice varieties have identified several metabolites or biomarkers against the gall midge biotype 1 (GMB1) pathogen using GC-MS (Agarrwal et al., 2014). Similarly, a number of metabolites were identified in rice crop in response to *Xanthomonas oryzae* pv. *oryzae* (Xoo; Sana et al., 2010). Another study demonstrated that the use of GC-MS could identify the accumulation of numerous metabolites in rice and barley crops against *Magnaporthe oryzae* (Parker et al., 2009). Meanwhile, phenylpropanoid and phenolic metabolites have been reported in wheat crop in response to biotic stress (Gunnaiah et al., 2012).

Metabolomics is particularly important in plant systems because plants produce more metabolites than either animals or microbes. The secondary metabolites produced by plants are helpful in responses to environmental stress. Thus, environmental metabolomics is a promising area in stress-physiology during that plant response to numerous abiotic stresses in relation to their metabolite changes (Brunetti et al., 2013; Viant and Sommer, 2013). Polyamine metabolites have been found in rice crop under drought stress conditions by applying GC-TOF-MS method (Do et al., 2013). In addition, a similar technique was used in rice, and the results identified salt tolerant cultivars (Liu et al., 2013; Gupta and De, 2017). Moreover, many metabolite analyses have been conducted in wheat, maize, tomato, and soybean crops in relation to drought, cold, and heat stress (Semel et al., 2007; Bowne et al., 2012; Silvente et al., 2012; Witt et al., 2012; Sun et al., 2016; Le et al., 2017; Paupiere et al., 2017). Several metabolomics techniques including LC/GC-MS, GC/EI-TOF-MS, HPLC, and NMR have been widely used in crop species such as, rice, tomato, maize and soybean in response to abiotic (drought, salt, oxidative, and temperature) and biotic stress conditions (Ghatak et al., 2018). Plant metabolite changes via certain pathways have been found to improve the nutritional value of genetically modified rice by the accumulation of β-carotene in the endosperm (Paine et al., 2005). Anthocyanin (a secondary metabolite) production was enhanced using metabolic engineering in tomato crops (Butelli et al., 2008). Therefore, metabolomics, coupled with other omics like genomics, transcriptomics, and proteomics, provide an integrated portrait of various functions ranging from the genome to metabolome as well as phenotypic characteristics (Weckwerth, 2011a). Strong correlations among these integrated omics have been identified in the responses of crops and plants to abiotic stress (Urano et al., 2010; Duque et al., 2013; Jogaiah et al., 2013).

### Ionomics

Ionomics deals with the ionome, whereas ionome refers to the total mineral nutrient and trace elemental composition and represents the cellular inorganic components of plant systems (Salt et al., 2008; Satismruti et al., 2013). Ionomics comprises the quantitative measurement of the elemental composition of an organism and identifies the changes in mineral composition triggered by various physiological stimuli, genetic modifications, or developmental conditions. It is a dynamic approach that is able to analyze the functions of genes and gene networks that characterize the ionome in response to physiological and environmental stress (Baxter, 2010). Ionomics acquisition by high throughput elemental profiling in plants has been conducted using different analytical tools including inductively coupled plasma-mass/optical emission spectroscopy (ICP-MS/OES), neutron activation analysis (NAA), and X-ray crystallography (Salt et al., 2008; Kumari et al., 2015). Inductively coupled plasma-mass spectroscopy technique is cheaper and can run hundreds of samples daily with excellent sensitivity to determine the elements. Nonetheless, ICP-OES may detect elements high throughput technique at the cost of some elements and sensitivity compared to ICP-MS. The XRF is faster to localize the elements but little cost than both ICP-MS/OES. Whereas, NAA is costly, slow and can detect more than 30 elements possible simultaneously (Salt et al., 2008).

In addition, the leaf ionome has been analyzed to identify the plant ionomic regulatory networks involved in iron and phosphorus homeostasis (Baxter, 2015). Using ICP-MS, the leaf and grain ionomes have been analyzed to generate genetic maps, identify QTLs, and detect mineral element genetic diversification in rice crop (Norton et al., 2010; Zhang et al., 2014b; Pinson et al., 2015).

Moreover, seed ionome analysis has found differential gene expression, improved symbiotic responses to mycorrhizal fungi, and altered growth phenotypes under phosphate starvation in maize crop (Mascher et al., 2014). Single seed-based ionomic profiling in maize crop may be influenced by environmental and genetic factors that affect seed ionome accumulation (Baxter et al., 2014). Comprehensive, elemental profiling has revealed the QTLs responsible for grain mineral accumulation and yield in maize crop (Gu et al., 2015). Additionally, one ionomics study elucidated the relationships and responses of elements, minerals, and metabolites in barley under salt stress (Wu et al., 2013). Following this, the ionomic screening was performed in mutant lines of soybean crop which altered seed ionomic composition. They determined elemental concentration by applying ICP-MS method (Ziegler et al., 2013). Elemental profiling analysis has also been conducted in tomato cultivars to observe the concentrations of micro- and macro-nutrients under water stress (Sanchez-Rodríguez et al., 2010). Similarly, ionomic profiling has been performed to analyze the nutrient balance in some fruit species including kiwifruits, oranges, mangos, apples, and blueberry (Parent et al., 2013). Therefore, those ionomic studies suggest the important role for crop improvement and responses to various abiotic and biotic stimuli.

In light of these results, the integration of ionomics with other omics, such as genomics or metabolomics, could serve to identify potential genes and their networks that improve crop resistance in response to physiological and environmental stress (Singh et al., 2013; Wu et al., 2013; Huang and Salt, 2016; Guo et al., 2017).

### Phenomics

Phenomics defined as the characterization of phenotypes through the acquisition of high-dimensional phenotypic data on an organism-wide scale (Houle et al., 2010). However, phenome refers to the phenotype as a whole and plant phenome can be determined by genome (G), environment (E), and management (M) interactions (Gjuvsland et al., 2013; Großkinsky et al., 2018), thus phenomenon is also referred to as genotype–phenotype–envirotype (G–P–E) interactions (Zhao et al., 2019).

Precise phenotyping is very accurate for the gene and QTL mapping of particular traits of interest to identify their roles via forward and reverse phenomics applications for the genetic improvement in crop plants (Kumar et al., 2015b). In both cases, the “best of the best” or the “best varieties of the best” germplasm lines could be detected through automated high-through put imaging technologies. These non-invasive imaging approaches allow for rapid phenotyping of the traits (phenes) through color imaging of the biomass, far infrared imaging of the canopy, lidar (light detection and ranging) to measure growth parameters, and magnetic resonance imaging to analyze root systems in crops (Finkel, 2009; Berger et al., 2010; Furbank and Tester, 2011). Furthermore, roots could be imaged in laboratories and greenhouses without damaging plant samples. For example electrical resistance tomography, electrical capacitance, X-ray computed tomography, and positron emission tomography were used to image root system for soil-grown plants and crops (McGrail et al., 2020). Red, green, blue (RGB) imaging, based on visible light, is a phenotyping tool used to estimate canopy and root systems (Großkinsky et al., 2018). Several studies have applied visible light imaging techniques through RGB set-ups in order to determine crop phenotyping parameters. For example, an RGB imaging setup scanned root system for *Triticum durum* grown in soil-filled rhizoboxes (Bodner et al., 2017), plant pthosystem, and disease symptoms were assessed through RGB color-based imaging (Mahlein, 2016) and RGB digital imaging method was used to analyze plant shoots phenotyping under various stress responses (Humplík et al., 2015). Using infrared thermography, one study confirmed the role of stomatal conductance under salinity stress in young barley and wheat seedlings (Sirault et al., 2009). Chlorophyll fluorescence imaging has also been applied to screen for abiotic stress responses in tobacco, canola, and cotton crops using pulse amplitude modulated (PAM) instruments (Saranga et al., 2004; Baker, 2008). Furthermore, digital imaging methods have quantified boron toxicity under abiotic stress in form of the mapping of wheat and barley populations (Schnurbusch et al., 2010). With regard to the responses to biotic stress, similar method has been applied to detect and quantify the disease symptoms caused by pathogens in barley crop (Swarbrick et al., 2006; Chaerle et al., 2009). Thus, phenomics applications may play a vital role in order to evaluate the phenotypic parameters in crops under biotic and abiotic stress conditions.

Phenotyping techniques are more important for analyzing crops in the field compared to plants in either the laboratory or greenhouse. Multi and hyperspectral technologies may be utilized to determine various agronomic characteristics (Rascher and Pieruschka, 2008). Among these, the laser-induced fluorescence transient (LIFT) technique is one of the most robust for analyzing the photosynthetic efficiency of crops in the field (Pieruschka et al., 2010). Access to wireless sensor a network aids in the phenotyping of crop traits and enables the accumulation of valuable data for breeding science (Ruiz-Garcia et al., 2009). Phenomics also offers various platforms connected with computational systems to analyze the phenotyping data including support vector machines (SVMs), artificial neural networks (ANNs), and principal component analysis (Karkee et al., 2009; Yang et al., 2009). So far, the major challenge in plant phenomics is to organize information systems to store datasets of phenotyping/traits so that they may be reanalyzed to generate new ideas (Cabrera-Bosquet et al., 2012).

The combination of GWAS and a high through-put rice phenotyping facility (HRPF) has resulted in the identification of 15 agronomic traits and 25 associated loci corresponding to the Green Revolution semi-dwarf gene (SD1) in rice (Yang et al., 2014). The multifunctional phenotyping technique was based on rice automatic phenotyping (RAP) and the yield traits scorer (YTS), which paved the way for high throughput phenotyping (HTP) methods to replace traditional phenotyping (manual phenotyping) approaches in crop breeding (Yang et al., 2014). Recently, an ontology-driven phenotyping hybrid information system (PHIS) has been proposed to assemble and share multi-scale data and metadata (Neveu et al., 2019). The ontology-driven PHIS information system is a powerful tool to integrate, manage data and share multi-source/multi-scale data (for both greenhouse and filed condition), however, ontology-driven architecture creates relationships between objects and enriches datasets with knowledge and metadata (Neveu et al., 2019). Current research efforts have also presented the Internet of Things (IoT)-based CropSight system, which is used to scale and determine both crop phenotyping and genotype–environment interactions (GxE). Internet of Things technologies is worldwide network which uses information and communications technologies for interconnection of sensing and actuating devices providing the ability to share information across platforms. This system can carry out high-quality crop phenotyping and monitor the dynamics of microclimate conditions and has been applied in field wheat crop experiments (Reynolds et al., 2019). However, Roitsch et al. (2019) proposed HTP applications with new generation sensors for next generation phenomics that would contribute to improving crop yields, stress tolerance, and management in the near future.

Overall, phenomics plays an important role in the development of crop breeding strategies through the integration of phenomics with other omics, such as genomics, proteomics, and metabolomics, to provide insights regarding the complex interactions between phenomes, the genome, and environmental factors, which will be beneficial for improving crop management.

## Role of Bioinformatics Within the Context of Databases and Software Tools for Crop Omics Analysis

Bioinformatics is an application of computational technology to handle and analyze the biological data. Bioinformatics as an interdisciplinary field encompasses computer science, statistics, mathematics, engineering, molecular biology, and biotechnology. Bioinformatics helps in order to interpret biological queries utilizing computational software (Raza et al., 2021). Notably, the integration of omics approaches (i.e., genomics, transcriptomics, proteomics, and metabolomics) has increased our understanding molecular processes associated with abiotic stress responses in plants (Cramer et al., 2011; Jogaiah et al., 2013). Nonetheless, bioinformatics consolidates with these omics approaches and provides base for collecting information for plant abiotic stresses (Ambrosino et al., 2020). Thus, bioinformatics is indispensable for data mining and organization (data production) in support of different omics technologies (Ambrosino et al., 2020). Furthermore, bioinformatics interprets information about the functional system of genes provided by such robust technologies. Bioinformatics also provides accessible resources for computational modeling and simulation analysis by integrating multiple omics technologies. The bioinformatics tools utilizing various software packages have been used for analyzing the multi-omics approaches in crop science. Recently, the availability and advancements of omics platforms have expanded remarkably to allow information to be utilized in multi-dimensional research in the plant sciences. The computational resources have not only made it possible to store, catalog, and analyze the available data but have also provided an easy means to access user friendly databases. Various multi-omics databases have been developed for the crop sciences (Table 1).

**Table 1:** List of online databases used for crop multi-omics analysis.

**S. no**.	**Database**	**Crop species**	**Features and functionality**	**Availability/URL**
1	Gramene	Multi-species	Multi-omics: comparative functional genomics, transcriptomics, and metabolic pathways	http://www.gramene.org/
2	Plant Reactome	Multi-species	Multi-omics: genomics, transcriptomics, functional proteomics, and integrated metabolic pathways	http://plants.reactome.org/
3	GabiPD	Multi-species	Multi-omics	http://www.gabipd.org/
4	KaPPA-View4 KEGG	Multi-species	Multi-omics: Metabolome integrated transcriptomic and genomic pathway	http://kpv.kazusa.or.jp/kpv4/kegg
5	KOMICS	Multi-species	Metabolomics	http://www.kazusa.or.jp/komics/en/
6	KNApSAcK	Multi- species	Metabolomics	http://kanaya.naist.jp/KNApSAcK/Family/
7	PMND	Multi-species	Multi-omics: Metabolome integrated transcriptome and genomic pathway	https://www.plantcyc.org/
8	PlantTFDB	Multi-species	Transcriptomics: Predicts plant transcription factors (TFs)	http://planttfdb.cbi.pku.edu.cn/
9	PPDB	Multi-species	Proteomics	http://ppdb.tc.cornell.edu/
10	GrainGenes	Multi-species	Genomics	http://www.graingenes.org
11	PlantGDB	Multi-species	Comparative genomics	http://www.plantgdb.org/
12	PCD	Multi-species	Genomics-assisted breeding (GAB)	https://www.pulsedb.org/
13	RAP-DB	Rice	Genomics integrated multi-omics	http://rapdb.dna.affrc.go.jp/
14	RiceXPro	Rice	Functional genomics and transcriptomics	http://ricexpro.dna.affrc.go.jp/
15	Ricebase	Rice	Genomics	https://ricebase.org/
16	Oryzabase	Rice	Integrated biological and genome information database	https://shigen.nig.ac.jp/rice/oryzabase/
17	SNP-Seek II	Rice	SNP-seek database	http://snp/seek.irri.org
18	RicyerDB	Rice	Integrative genomics and proteomics	http://server.malab.cn/Ricyer/index.html
19	RiceVarMap	Rice	Genomic variation and functional annotation	http:/ricevarmap.ncpgr.cn
20	TFGD	Tomato	Functional genomics, integrated transcriptomics, and metabolomics	http://ted.bti.cornell.edu/
21	TOMATOMICS	Tomato	Multi-omics	http://plantomics.mind.meiji.ac.jp/tomatomics/index.html
22	SoyKB	Soybean	Multi-omics	http://soykb.org/
23	SoyBase	Soybean	Multi-omics	https://soybase.org/
24	SoyGD	Soybean	Genomics	http://soybeangenome.siu.edu/
25	SFGD	Soybean	Multi-omics: Functional genomics, transcriptomics, and metabolic pathways	http://bioinformatics.cau.edu.cn/SFGD/
26	SoyNet	Soybean	Functional genomics and transcriptomics	https://www.inetbio.org/soynet/
27	MaizeGDB	Maize	Multi-omics	https://www.maizegdb.org/
28	MaizeDIG	Maize	Phenomics and genomics	https://maizedig.maizegdb.org/
29	MaizeSNPDB	Maize	SNPs	https://github.com/venyao/MaizeSNPDB
30	MMAD	Maize	Microarray	http://maizearrayannot.bi.up.ac.za/
31	CSRDB	Maize and rice	Small RNAs database	http://sundarlab.ucdavis.edu/smrnas/
32	RGPDB	Maize, soybean and sorghum	Multi-omics	http://sysbio.unl.edu/RGPDB/
33	CerealsDB	Wheat	Functional genomics	http://www.cerealsdb.uk.net/cerealgeno/mics/
34	WGI	Wheat	Genomics	http://wheatgenome.info/
35	wDBTF	Wheat	Transcription factors	http://wwwappli.nantes.inra.fr:8180/wDBFT/
36	WheatGPE	Wheat	Base on phenotype-genotype and environment	http://www.wheatdb.org/
37	SiFGD	Foxtail millet	Functional genomics integrated transcriptomics, and metabolomics	http://structuralbiology.cau.edu.cn/SIFGD/
38	CottonFGD	Cotton	Functional genomics	https://cottonfgd.org/
39	CottonGen	Cotton	Genomics	https://www.cottongen.org/
40	CottonQTLdb	Cotton	QTL analysis	http://www.cottonqtldb.org
41	MTGD	*Medicago truncatula*	Genomics	http://www.MedicagoGenome.org
42	CTDB	Chickpea	Functional genomics and transcriptomics	http://www.nipgr.ac.in/ctdb.html
43	ECPD	Potato	Genomics	https://www.europotato.org/
44	BARLEX	Barley	Genomics	http://barlex.barleysequence.org
45	SorghumFDB	Sorghum	Functional genomics	http://structuralbiology.cau.edu.cn/sorghum/index.html
46	SorGSD	Sorghum	SNPs	http://sorgsd.big.ac.cn/
47	SGH	Sugarcane	Genomics	https://sugarcane-genome.cirad.fr/
48	BRAD	*Brassica* species	Genomics and transcriptomics	http://brassicadb.org/brad/
49	CropSNPdb	*Brassica* species and wheat	SNPs	http://snpdb.appliedbioinformatics.com.au/
50	SFGD	Sunflower	Genomics and transcriptomics	https://www.sunflowergenome.org
51	BBDG	Blueberry	Genomics and transcriptomics	http://bioinformatics.towson.edu/BBGD/

*GABI, genomanalyse im biologischen system pflanze; GabiPD, GABI primary database; PMND, plant metabolic network database; SiFGD, Setaria italica functional genomics database; RAP-DB, the rice annotation project database; TFGD, tomato functional genomics database; TFDB, trancription factors database; PPDB, plant proteome database; PCD, pulse crop database; RiceVarMap, rice variation map; SoyGD, soybean genome database; SFGD, soybean functional genomics database; SoyNet, soybean network; MaizeGDB, maize genetics and genomics database; MaizeDIG, database of images and genomes; MMAD, maize microarray annotation database; CSRDB, cereal small RNAs database; RGPDB, root genes and promoters database; WGI, wheat genome info; wDBTF, wheat database for transcription factor; WheatGPE, Genotype-phenotype and environment; CottonFGD, CottonFGD cotton functional genomics database; CottonGen, cotton genomics; MtGD, Medicago truncatula genome database; CTDB, chickpea transcriptome database; ECPD, European cultivated potato database; SorghumFDB, Sorghum functional genomics database; SorGSD, Sorghum genome SNP database; SGH, sugarcane genome hub; BRAD, (Brassica database; SFGD, sunflower genome database; BBDG, blueberry genomics database*.

Among these, Gramene, Plant Reactome, GabiPD, KaPPA-View4 KEGG, and PMND provide multi-omics-based integration of genomics, transcriptomics, proteomics, and metabolomics for several crop species. Both KNApSAcK and KOMICS are useful metabolomics databases that provide information on abundant metabolites in medical plants and crop species. The KOMICS contains several databases for metabolome analysis for example Food Metabolome Repository that can be used to obtain data from various Japanese foods using liquid chromatography-mass spectrometry (LC-MS), KomicMarket database which is used for detected peaks (known/unknown) in metabolome analysis. Similarly Metabolonote database available at KOMICS can also be used to manage “metadata” for experimental 616 data obtained through the metabolomics studies.

PlantTFDB is a multi-crop species database that predicts plant TFs. Moreover, single-crop species dedicated databases of important crops are also available, such as RAP-DB (rice), TFGD (tomato), SoyKB (soybean), MaizeGDB (maize), CerealsDB (wheat), RiceXPro (rice), and SiFGD (Foxtail millet; Table 1). These databases provide comprehensive data on functional genomics coupled with transcriptomics, proteomics, and metabolomics and are currently playing pivotal roles in breeding sciences. Furthermore, there are more than 50 databases numerous crops that provide accumulated omics analysis data (for detailed information see Table 1).

Various software packages have also been developed for multi-omics analysis. In this regard, various online tools have been compiled and are presented in Table 2. The software packages are important to analyze the phenotyping, measurement, and disease symptoms of leaf such as, BioLeaf and EasyPCC. Whereas, protein–protein interaction, gene structure analysis can also predicted in STRING and GSDS, respectively. However, Gromacs software could be used for simulation of protein and lipids for crops (for detail see Table 2).

**Table 2:** List of online software packages used for crop multi-omics analysis.

**S. no**.	**Software**	**Crop species**	**Features and functionality**	**Availability/URL**
1	LemnaLauncher	Multi-species	Phenomics: image-based measurements of length, width, color, surface of seeds	http://www.plant/image/analysis.org/software/lemnalauncher
2	EasyPCC	Multi-species	Phenomics: field crop canopy measurement	http://www.plant/image/analysis.org/software/easypcc
3	BioLeaf	Multi-species	Phenomics: leaf surface and disease analysis	http://www.plant/image/analysis.org/software/bioleaf
4	STRING	Multi-species	Proteomics: predicts protein interactions containing functional associations	http://string.embl.de
5	SPPS	Multi-species	Proteomics: predicts protein–protein interaction partners	http://mdl.shsmu.edu.cn/SPPS/
6	ProteinProspector	Multi-species	Proteomics: sequence mining with MS	http://prospector.ucsf.edu/
7	Gromacs	Multiple-species	Genomics, proteomics and metabolomics	https://omictools.com/gromacs/tool
8	PTools	Multi-species	Multi-omics	https://omictools.com/ptools/tool
9	GSDS	Multi-species	Structural genomics: visualizes gene structure (exons, introns, and UTRs).	http://gsds.cbi.pku.edu.cn/
10	GAP4	Multi-species	Structural genomic sequence assembly	http://stadensourceforge.net/overview.html
11	VISTA	Multi-species	Comparative genomics	http://genome.lbl.gov/vista/index.shtml
12	AMDIS	Multi-species	Metabolomics: GC-MS data interpretation	http://www.amdis.net/
13	SIMCA-P 14.0	Multi-species	Ionomics integrated with metabolome: principal metabolic component analysis	https://umetrics.com/kb/simca/online/140

*SPPS, sequence-based protein partners search, GAP4, genome assembly program; GSDS, gene structure display server*.

Several softwares can be used as individual omics analysis such as, phenomics, proteomics, and metabolomics, whereas, some of them are useful for multi-omics analysis. In detail, LemnaLauncher, BioLeaf, and EasyPCC are used for phenomics analysis of crops. For proteome function and interactions, three important software packages (i.e., STRING, SPPS, and ProteinProspector) provide vital information. Importantly, Gromacs and PTools are multi-omics based software for multiple crop species. In order to analyze structural and comparative genomics, GSDS, GAP4, and VISTA are accessible tools for crop omics. Additionally, AMDIS and SIMCA-P 14.0 could be used for ionome-integrated metabolic component analysis in crop species (Table 2).

## Role of Panomics for Crop Breeding Science

Panomics provides a platform to integrate complex omics, such as genomics, epigenomics, transcriptomics, proteomics, PTM proteomics, metabolomics, and phenomics (Weckwerth et al., 2020). The concept of panomics was recently proposed by Weckwerth et al. (2020). The idea of this platform is to combine different omics and construct models that can be used to predict complex traits (Weckwerth, 2011a, 2019). However, coupling phenomics and environmental information with genomics, transcriptomics, proteomics, and metabolomics would provide a better understanding of the terroir-phenotype dependency at a molecular level. The integration of complex “omics” datasets could also reduce the number of false positives generated from single data sources for genotype-phenotype prediction (Ritchie et al., 2015). Panomics and environmental platforms together with multiple data integration can be used to identify genes, QTLs, and markers through functional omics and mathematical models to enhance the tolerance to abiotic and biotic stress in crop varieties and create elite lines to improve the germplasm (Weckwerth et al., 2020). To analyze the integrated data, special tools can be used to merge multi-omics datasets prior to any interpretation (Kuo et al., 2013) (e.g., tools such as PAINTOMICS, KaPPA-view, and COVAIN). PAINTOMICS is web based tool offers integrated visualization of data of two omics, the transcriptomics and metabolomics datasets and displays the data on KEGG pathway maps (García-Alcalde et al., 2011). Another web based tool the KaPPA-view has been developed for integration of transcript and metabolite data on plant metabolic pathway maps (Tokimatsu et al., 2005). Nevertheless, the COVAIN (covariance inverse) tool primarily used for metabolomics data and can support in statistical analysis of the integrated omics dataset with KEGG pathway and gene ontology analysis (Sun and Weckwerth, 2012). The integration of GWAS with panomics has also been used to explain and understand phenotypic variance in crops. Importantly, integrating GWAS with omics datasets including transcriptomics (eQTLS), proteomics (pQTLS), and metabolomics (mQTLS) may lead to the identification of novel genes and functional pathways underlying complex traits (Weckwerth et al., 2020). In this vein, a combined metabolome-based genome-wide association study (mGWAS with eQTL) identified metabolite features associated with kernel weight in maize crop (Wen et al., 2014).

Furthermore, the integration of panomics and genome editing tools (e.g., TALENs and CRISPR/Cas9) has been proposed as a model for the development of precision breeding (Weckwerth et al., 2020). Recently, using MAS and genomic selection techniques, agronomically important genes have been identified that only explain ~40% of the phenotypic variance. Hence, the proposed methodology of the integration of panomics with genome editing tools could result in the identification of the remaining ~60% of the phenotypic variance and may support the identification of agronomically important genes in a fast and effective manner to support precision breeding efforts. Thus, this methodology will not only be helpful for improving crops but will also ensure precision in trait optimization in terms of yield, nutritional value, and plant fitness (Weckwerth et al., 2020). Hence, genotype to phenotype concept based on epigenetic regulation (triggered by environmental factors) through integration of mutli-omics could lead to develop qualitative and quantitative traits which may be helpful for crop breeding improvement (Figure 2).

## Integration of Multi-Omics and Systems Biology Approaches for Crop Improvement

In order to understand the cellular components and complex behaviors of biological systems, an integration of the different omics approaches is required to envisage the responses of a given organism under a set of conditions. Previously, the coupling of metabolomics with genomics, transcriptomics, and proteomics provided an integrated portrait of functions, spanning the genome to phenotypic interactions with the environment (Weckwerth, 2011a). Combined omics approaches have been applied in potato tubers and Arabidopsis to analyze transcriptomic and metabolomic profiles (Urbanczyk-Wochniak et al., 2003; Hirai et al., 2004). These studies have demonstrated that coupling of different omics approaches could be useful for identifying potential candidate genes for functional analysis. Since the advancements in omics technologies and computational tools, integrative omics approaches have been implemented in the crop sciences. For example, the epigenetic-based integration of multi-omics revealed the role of the regulation of lipid biosynthesis during cotton fiber development (Wang et al., 2016). The integration of GWAS with metabolite profiling strategies has proved to be a powerful technique to dissect the biochemical and genetic processes in several model crop species including rice, maize, and tomato (Luo, 2015; Matsuda et al., 2015). Importantly, the integration of omics approaches (i.e., genomics, transcriptomics, proteomics, and metabolomics) has led to abiotic stress tolerant crop phenotypes (Jogaiah et al., 2013). Functional genomics and mutagenomics have been used to identify numerous mutants with specific variations with regard to growth, development, and stress tolerance in various crops including rice, maize, wheat, and barley (Talukdar and Sinjushin, 2015). Combined GWAS and HRPF approach was able to elucidate agronomic traits responsible for biomass growth and yield in rice crop (Yang et al., 2014). This robust technique replaced the traditional phenomics, providing a powerful tool for crop genetics and breeding sciences (Yang et al., 2014). Combined GWAS and high-throughput leaf scoring (HLS) was used to identify new loci related to the size, shape, and color of leaves in rice crop (Yang et al., 2015). The performance of QTL mapping combined with agronomic traits also helped to identify numerous QTLs in maize crop (Zhang et al., 2017). Hence, genomic information combined with potential phenotyping approaches can provide information on complex traits to improve crops (Zhao et al., 2019). Combined omics approaches could complement each other when analyzing certain biological processes. This idea has been validated through the differential regulation of metabolites, proteins, and ions related to salinity stress in halophytes (Kumari et al., 2015). Metabolomics is considered to be a link between genotypes and phenotypes (Fiehn, 2002). Combined ionome and metabolome techniques were used to suppress photosynthesis and growth rates in maize crop under alkaline conditions (Guo et al., 2017). Similarly, the leaf and grain ionome revealed mineral element genetic diversification in rice crop through genetic mapping and QTL identification (Norton et al., 2010; Zhang et al., 2014b; Pinson et al., 2015). Thus, genotype to phenotype-based integration of multi-omics would provide insights into the functional mechanisms of genes and their networks to improve crop science, genetics, growth, yield, and resistance in response to physiological and environmental stress (Figure 2).

Systems biology attempts to understand the complete biological system through modeling. It predicts the behavior of all components and interactions among genes, proteins, and metabolites with respect to external stimuli (Kumar et al., 2015a). Systems biology has provided a powerful base to combine multi-omics to create a holistic understanding of an organism related to its adaptation and development (Pinu et al., 2019). Multi-omics approaches have been employed in plant stress research associated with systems biology (Mosa et al., 2017). However, comprehensive analyses using three omics technologies, transcriptomics, metabolomics, and proteomics, have also increased our understanding of systems biology associated with abiotic stress responses in plants (Cramer et al., 2011). Multi-omics integrated with systems biology based on top-down and bottom-up data reduction approaches, which employ genomics and/or metabolomics as a foundation, is able to predict phenotypic responses and metabolic pathways (Pinu et al., 2019). Another study proposed two system-based approaches for decoding the complexity of biological systems. First, top-down or integrative systems biology has been employed with high-throughput multi-omics data and data analysis using bioinformatics and systems biology tools to identify agriculturally important traits. Second, bottom-up or predictive systems biology in which the properties of genes or proteins with available quantitative information are utilized to develop models of well-characterized components of both genes and proteins has been used to predict the behavior of systems under different conditions (Kumar et al., 2015a). Hence, a model needs to be developed and linked to phenotypic traits to allow for valuable progress with regard to genetic manipulation and crop production. The integration of multi-omics and systems biology approaches has resulted in the identification of molecular regulator networks for salt stress tolerance in grapevine crop (Daldoul et al., 2014). Moreover, systems biology integrated with omics approaches for network and testing models has been proposed for abiotic stress responses in crop plants (Gupta et al., 2013). In this regard, we proposed top-down (phenotype to genotype) and bottom-up (genotype to phenotype) model based on an integration of multi-omics with systems biology in response to environmental stress, which may also be useful to improve crop breeding (Figure 2).

## Conclusion and Perspective

Multi-omics analysis has played an integral role in the identification of genetic processes, growth, development, and stress tolerance in various crops. Several omics approaches including genomics, transcriptomics, proteomics, metabolomics, ionomics, and phenomics have employed high throughput techniques to interpret functional analysis, molecular mechanisms of genes, and gene networks in crop science. Furthermore, the integration of GWAS with metabolomics, transcriptomics, and proteomics has proved to be a potential tool to elucidate biochemical processes and abiotic stress tolerance in some model crops. The studies have shown that how the combination of several omics approaches could be beneficial for identifying potential candidate genes and their pathways. With advances in high throughput technologies and computational tools, the integration of some omics approaches has been possible in the crop sciences. The panomics platform with integrated multi-omics, such as genomics, epigenomics, transcriptomics, proteomics, proteomics, metabolomics, and phenomics, would facilitate the construction of models to predict agronomically important traits to improve crops through precision breeding. Importantly, the integration of systems biology with complex omics datasets has also increased our understanding of molecular regulator networks for crop improvement. The studies have revealed the G–P–E interactions in crops. Subsequently, integration of functional genomics with trancriptomics, proteomics, metabolomics, and ionomics may result in apparent crop quality phenotypic traits under certain stresses through “genotype to phenotype” concept. From this perspective, we propose, the integration of multi-omics with systems biology by top-down (phenotype to genotype) and bottom-up (genotype to phenotype) model that can be helpful to develop quality agronomic traits for crop improvements under environmental stress conditions (Figure 2).

## Author Contributions

YY, MS, WA, YW, and JZ drafted the manuscript. YY, JZ, JL, and MS collected the background information. MHS and FW analyzed the databases and software tools for omics. All authors read and approved the final manuscript.

## Conflict of Interest

The authors declare that the research was conducted in the absence of any commercial or financial relationships that could be construed as a potential conflict of interest.

## Publisher's Note

All claims expressed in this article are solely those of the authors and do not necessarily represent those of their affiliated organizations, or those of the publisher, the editors and the reviewers. Any product that may be evaluated in this article, or claim that may be made by its manufacturer, is not guaranteed or endorsed by the publisher.

## References

[B1] Abdelrahman M. El-Sayed M. A. Hashem A. Abd-Allah E. F. Alqarawi A. A. Burritt D. J.. (2018). Metabolomics and transcriptomics in legumes under phosphate deficiency in relation to nitrogen fixation by root nodules. Front. Plant Sci. 9:922. 10.3389/fpls.2018.00922 30050543 PMC6052890

[B2] Agarrwal R. Bentur J. S. Nair S. (2014). Gas chromatography mass spectrometry based metabolic profiling reveals biomarkers involved in rice-gall midge interactions. J. Integr. Plant. Biol. 56, 837–848. 10.1111/jipb.12244 25059749

[B3] Agarwal M. Shrivastava N. Padh H. (2008). Advances in molecular marker techniques and their applications in plant sciences. Plant Cell Rep. 27, 617–631. 10.1007/s00299-008-0507-z 18246355

[B4] Ahloowalia B. S. Maluszynski M. Nichterlein K. (2004). Global impact of mutation derived varieties. Euphytica 135, 187–204. 10.1023/B:EUPH.0000014914.85465.4f

[B5] Aizat W. M. Hassan M. (2018). Proteomics in systems biology, in Omics Applications for Systems Biology. Advances in Experimental Medicine and Biology, eds Aizat W. Goh H. H. Baharum S. (Cham: Springer), 31–49. 10.1007/978-3-319-98758-3_3 30382567

[B6] Allwood J. W. Goodacre R. (2010). An introduction to liquid chromatography-mass spectrometry instrumentation applied in plant metabolomic analyses. Phytochem. Anal. 21, 33–47. 10.1002/pca.1187 19927296

[B7] Alseekh S. Aharoni A. Brotman Y. Contrepois K. D'Auria J. Ewald J.. (2021). Mass spectrometry-based metabolomics: a guide for annotation, quantification and best reporting practices. Nat. Methods 18, 747–756. 10.1038/s41592-021-01197-1 34239102 PMC8592384

[B8] Ambrosino L. Colantuono C. Diretto G. Fiore A. Chiusano M. L. (2020). Bioinformatics resources for plant abiotic stress responses: state of the art and opportunities in the fast evolving-omics era. Plants 9:591. 10.3390/plants9050591 32384671 PMC7285221

[B9] Appleby N. Edwards D. Batley J. (2009). New technologies for ultra-high throughput genotyping in plants, in Methods in Molecular Biology, Plant Genomics, eds Gustafson J. P. Langridge P. Somers D. J. Totowa N. J. (New York, NY: Humana Press), 19–39. 10.1007/978-1-59745-427-8_2 19347650

[B10] Arefian M. Vessal S. Malekzadeh-Shafaroudi S. Siddique K. H. M. Bagheri A. (2019). Comparative proteomics and gene expression analyses revealed responsive proteins and mechanisms for salt tolerance in chickpea genotypes. BMC Plant Biol. 19:300. 10.1186/s12870-019-1793-z 31288738 PMC6617847

[B11] Aslam B. Basit M. Atif N. M. Khurshid M. Rasool M. H. (2017). Proteomics: technologies and their applications. J. Chromato Sci. 55,182–196. 10.1093/chromsci/bmw167 28087761

[B12] Baggerman G.eert. Vierstraete E. De L. A. Schoofs L. (2005). Gel-based versus gel-free proteomics: a review. Comb. Chem. High Throughput Screen. 8, 669–677. 10.2174/138620705774962490 16464155

[B13] Baharum S. N. Azizan K. A. (2018). Metabolomics in systems biology. Adv. Exp. Med.Biol. 1102, 51–68. 10.1007/978-3-319-98758-3_4 30382568

[B14] Baker N. R. (2008). Chlorophyll fluorescence: a probe of photosynthesis *in vivo*. Annu. Rev. Plant Biol. 59, 89–113. 10.1146/annurev.arplant.59.032607.092759 18444897

[B15] Balmerl D. Flors V. Glauser G. Mauch-Mani B. (2013). Metabolomics of cereals under biotic stress: current knowledge and techniques. Front. Plant Sci. 4:82. 10.3389/fpls.2013.00082 23630531 PMC3632780

[B16] Basha S. M. Mazhar H. Vasanthaiah H. K. N. (2010). Proteomics approach to identify unique xylem sap proteins in Pierce's disease-tolerant *Vitis* species. Appl. Biochem. Biotechnol. 160, 932–944. 10.1007/s12010-009-8620-1 19412582

[B17] Bashir K. Matsui A. Rasheed S. Seki M. (2019). Recent advances in the characterization of plant transcriptomes in response to drought, salinity, heat, and cold stress. F1000Res. 8:658. 10.12688/f1000research.18424.1 31131087 PMC6518435

[B18] Baxter I. (2010). Ionomics: the functional genomics of elements. Brief. Funct. Genomics 9, 149–156. 10.1093/bfgp/elp055 20081216

[B19] Baxter I. (2015). Should we treat the ionome as a combination of individual elements, or should we be deriving novel combined traits? J. Exp. Bot. 66, 2127–2131. 10.1093/jxb/erv040 25711709 PMC4986723

[B20] Baxter I. R. Ziegler G. Lahner B. Mickelbart M. V. Foley R. Danku J.. (2014). Single-kernel ionomic profiles are highly heritable indicators of genetic and environmental influences on elemental accumulation in maize grain (*Zea mays*). PLoS ONE 9:e87628. 10.1371/journal.pone.0087628 24489944 PMC3906179

[B21] Berger B. Parent B. Tester M. (2010). High-throughput shoot imaging to study drought responses. J. Exp. Bot. 61, 3519–3528. 10.1093/jxb/erq201 20660495

[B22] Bhardwaj A. R. Joshi G. Kukreja B. Malik V. Arora P. Pandey R.. (2015). Global insights into high temperature and drought stress regulated genes by RNA-Seq in economically important oilseed crop *Brassica juncea*. BMC Plant Biol. 15:9. 10.1186/s12870-014-0405-1 25604693 PMC4310166

[B23] Bodner G. Alsalem M. Nakhforoosh A. Arnold T. Leitner D. (2017). RGB and spectral root imaging for plant phenotyping and physiological research: experimental setup and imaging protocols. J Vis Exp. 126:56251. 10.3791/56251 28809835 PMC5614140

[B24] Bonthala V. S. Mayes K. Moreton J. Blythe M. Wright V. May S. T.. (2016). Identification of gene modules associated with low temperatures response in Bambara groundnut by network-based analysis. PLoS ONE 11:e0148771. 10.1371/journal.pone.0148771 26859686 PMC4747569

[B25] Bowne J. B. Erwin T. A. Juttner J. Schnurbusch T. Langridge P. Bacic A.. (2012). Drought responses of leaf tissues from wheat cultivars of differing drought tolerance at the metabolite level. Mol. Plant 5, 418–429. 10.1093/mp/ssr114 22207720

[B26] Brunetti C. George R. M. Tattini M. Field K. Davey M. P. (2013). Metabolomics in plant environmental physiology. J. Exp. Bot. 64, 4011–4020. 10.1093/jxb/ert244 23922358

[B27] Burgess D. J. (2015). Putting transcriptomics in its place. Nat. Rev. Genet. 16:319. 10.1038/nrg3951 25948245

[B28] Butelli E. Titta L. Giorgio M. Mock H. P. Matros A. Peterek S.. (2008). Enrichment of tomato fruit with health-promoting anthocyanins by expression of select transcription factors. Nat. Biotechol. 26, 1301–1308. 10.1038/nbt.1506 18953354

[B29] Cabrera-Bosquet L. Crossa J. von Zitzewitz J. Serret M. D. Araus J. L. (2012). High-throughput phenotyping and genomic selection: the frontiers of crop breeding converge. J. Integ. Plant. Biol. 54, 312–320. 10.1111/j.1744-7909.2012.01116.x 22420640

[B30] Caldwell D. G. McCallum N. Shaw P. Muehlbauer G. J. Marshall D. F. Waugh R. (2004). A structured mutant population for forward and reverse genetics in barley (*Hordeum vulgare* L.). Plant J. 40, 143–150. 10.1111/j.1365-313X.2004.02190.x 15361148

[B31] Callinan P. A. Feinberg A. P. (2006). The emerging science of epigenomics. Human Mol. Genet. 15 (Suppl_1), R95–R101. 10.1093/hmg/ddl095 16651376

[B32] Campbell B. C. Gilding E. K. Mace E. S. Tai S. Tao Y. Prentis P. J.. (2016). Domestication and the storage starch biosynthesis pathway: signatures of selection from a whole sorghum genome sequencing strategy. Plant Biotechnol. J. 14, 2240–2253. 10.1111/pbi.12578 27155090 PMC5103234

[B33] Cao A. H. Xing L. P. Wang X. Y. Yang X. M. Wang W. YSun Y. L.. (2011). Serine/threonine kinase gene Stpk-V, a key member of powdery mildew resistance gene Pm21, confers powdery mildew resistance in wheat. Proc. Natl. Acad. Sci. U.S.A. 108, 7727–7732. 10.1073/pnas.1016981108 21508323 PMC3093467

[B34] Chaerle L. Lenk S. Leinonen I. Jones H. G. Van Der Straeten D. Buschmann C. (2009). Multi-sensor plant imaging: towards the development of a stress-catalogue. Biotechnol. J. 4, 1152–1167. 10.1002/biot.200800242 19557794

[B35] Challa S. Neelapu N. R. (2018). Genome-wide association studies (GWAS) for abiotic stress tolerance in plants, in Biochemical, Physiological and Molecular Avenues for Combating Abiotic Stress Tolerance in Plants, eds Shabir S. H. Hussain Wani (Cham: Academic Press), 135–150. 10.1016/B978-0-12-813066-7.00009-7

[B36] Chekanova J. A. (2015). Long non-coding RNAs and their functions in plants. Curr. Opin. Plant Biol. 27, 207–216. 10.1016/j.pbi.2015.08.003 26342908

[B37] Chen H. Wang L. Liu X. Hu L. Wang S. Cheng X. (2017). *De novo* transcriptomic analysis of cowpea (*Vigna unguiculata* L. Walp.) for genic SSR marker development. BMC Genet. 18:65. 10.1186/s12863-017-0531-5 28693419 PMC5504845

[B38] Chen L. Hao L. Parry M. A. J. Phillips A. L. Hu Y. G. (2014). Progress in TILLING as a tool for functional genomics and improvement of crops. J. Integr. Plant Biol. 56, 425–443. 10.1111/jipb.12192 24618006

[B39] Cokus S. J. Feng S. Zhang X. Chen Z. Merriman B. Haudenschild C. D.. (2014). Shotgun bisulphite sequencing of the Arabidopsis genome reveals DNA methylation patterning. Nature 452, 215–219. 10.1038/nature06745 18278030 PMC2377394

[B40] Cooper J. L. Till B. J. Laport R. G. Darlow M. C. Kleffner J. M. Jamai A.. (2008). TILLING to detect induced mutations in soybean. BMC Plant Biol. 8:9. 10.1186/1471-2229-8-9 18218134 PMC2266751

[B41] Cramer G. R. Urano K. Delrot S. Pezzotti M. Shinozaki K. (2011). Effects of abiotic stress on plants: a systems biology perspective. BMC Plant Biol. 11:163. 10.1186/1471-2229-11-163 22094046 PMC3252258

[B42] Daldoul S. Ben Amar A. Guillaumie S. Mliki A. (2014). Integration of omics and system biology approaches to study grapevine (*Vitis vinifera* L.) response to salt stress: a perspective for functional genomics - a review. OENO One 48, 189–200. 10.20870/oeno-one.2014.48.3.1573

[B43] De Cremer K. Mathys J. Vos C. Froenicke L. Michelmore R. W. Cammue B. P. A.. (2013). RNAseq-based transcriptome analysis of *Lactuca sativa* infected by the fungal necrotroph *Botrytis cinerea*. Plant Cell Environ. 36, 1992–2007. 10.1111/pce.12106 23534608

[B44] Deeba F. Pandey A. K. Ranjan S. Mishra A. Singh R. Sharma Y. K.. (2012). Physiological and proteomic responses of cotton (*Gossypium herbaceum* L.) to drought stress. Plant Physiol. Biochem. 53, 6–18. 10.1016/j.plaphy.2012.01.002 22285410

[B45] Demirevska K. Simova-Stoilova L. Vassileva V. Vaseva I. Grigorova B. Feller U. (2008). Drought-induced leaf protein alterations in sensitive and tolerant wheat varieties. Gen. Appl. Plant Physiol. 34, 79–102. 10.7892/boris.110728

[B46] Deshmukh R. Sonah H. Patil G. Chen W. Prince S. Mutava R.. (2014). Integrating omic approaches for abiotic stress tolerance in soybean. Front. Plant Sci. 25:244. 10.3389/fpls.2014.00244 24917870 PMC4042060

[B47] Di-Baccio D. Navari-Izzo F. Izzo R. (2004). Seawater irrigation: antioxidant defence responses in leaves and roots of a sunflower (*Helianthus annuus* L.) ecotype. J. Plant Physiol. 161, 1359–1366. 10.1016/j.jplph.2003.07.001 15658806

[B48] Ding X. Li X. Xiong L. (2013). Insight into differential responses of upland and paddy rice to drought stress by comparative expression profiling analysis. Int. J. Mol. Sci. 14, 5214–5238. 10.3390/ijms14035214 23459234 PMC3634487

[B49] Do P. T. Degenkolbe T. Erban A. Heyer A. G. Kopka J. Kohl K. I.. (2013). Dissecting rice polyamine metabolism under controlled long-term drought stress. PLoS ONE 8:e60325. 10.1371/journal.pone.0060325 23577102 PMC3620119

[B50] Dong C. Dalton-Morgan J. Vincent K. Sharp P. (2009). A modified tilling method for wheat breeding. Plant Gen. 2, 39–47. 10.3835/plantgenome2008.10.0012

[B51] Dugas D. V. Monaco M. K. Olsen A. Klein R. R. Kumari S. Ware D.. (2011). Functional annotation of the transcriptome of *Sorghum bicolor* in response to osmotic stress and abscisic acid. BMC Genomics 12:514. 10.1186/1471-2164-12-514 22008187 PMC3219791

[B52] Duque A. S. Almeida A. M. Bernardes da Silva A. Marques da Silva, J. Farinha A. P. Santos D.. (2013). Chapter 3: Abiotic stress responses in plants: unraveling the complexity of genes and networks to survive, in Abiotic Stress: Plant Responses and Applications in Agriculture, eds Vahdati K. Leslie C. (Rijeka: INTECH Open), 49–102. 10.5772/45842

[B53] Dwivedi S. Perotti E. Ortiz R. (2008). Towards molecular breeding of reproductive traits in cereal crops. Plant Biotechnol. J. 6, 529–559. 10.1111/j.1467-7652.2008.00343.x 18507792

[B54] Eldakak M. Milad S. I. Nawar A. I. Rohila J. S. (2013). Proteomics: a biotechnology tool for crop improvement. Front. Plant Sci. 4:35. 10.3389/fpls.2013.00035 23450788 PMC3584254

[B55] El-Metwally S. Ouda O. M. Helmy M. (2014). Next Generation Sequencing Technologies and Challenges in Sequence Assembly, 1st Edn. New York, NY: Springer. 10.1007/978-1-4939-0715-1

[B56] Fiehn O. (2002). Metabolomics — the link between genotypes and phenotypes. Plant Mol. Biol. 48, 155–171. 10.1023/A:1013713905833 11860207

[B57] Fiehn O. Kopka J. Dormann P. Altmann T. Trethewey R. N. Willmitzer L. (2000). Metabolite profiling for plant functional genomics. Nat. Biotechnol. 18, 1157–1161. 10.1038/81137 11062433

[B58] Finkel E. (2009). With ‘phenomics’ plant scientists hope to shift breeding into overdrive. Science 325, 380–381. 10.1126/science.325_380 19628831

[B59] Fournier M. L. Gilmore J. M. Martin-Brown S. A. Washburn M. P. (2007). Multidimensional separations-based shotgun proteomics. Chem. Rev. 107, 3654–3686. 10.1021/cr068279a 17649983

[B60] Francia E. Morcia C. Pasquariello M. Mazzamurro V. Milc J. A. Rizza F.. (2016). Copy number variation at the HvCBF4-HvCBF2 genomic segment is a major component of frost resistance in barley. Plant Mol. Biol. 92. 161–175. 10.1007/s11103-016-0505-4 27338258

[B61] Fukuoka S. Saka N. Koga H. Ono K. Shimizu T. Ebana K.. (2009). Loss of function of a proline-containing protein confers durable disease resistance in rice. Science 325, 998–1001. 10.1126/science.1175550 19696351

[B62] Furbank R. T. Tester M. (2011). Phenomics—technologies to relieve the phenotyping bottleneck. Tred. Plant Sci. 16, 635–644. 10.1016/j.tplants.2011.09.005 22074787

[B63] Gabur I. Chawla H. S. Liu X. Kumar V. Faure S. von Tiedemann S.. (2018). Finding invisible quantitative trait loci with missing data. Plant Biotechnol. J. 16, 2102–2112. 10.1111/pbi.12942 29729219 PMC6230954

[B64] García-Alcalde F. García-López F. Dopazo J. Conesa A. (2011). Paintomics: a web based tool for the joint visualization of transcriptomics and metabolomics data. Bioinformatics 27, 137–139. 10.1093/bioinformatics/btq594 21098431 PMC3008637

[B65] Gent J. I. Ellis N. A. Guo L. Harkess A. E. Yao Y. Zhang X.. (2013). CHH islands: *de novo* DNA methylation in near-gene chromatin regulation in maize. Genome Res. 23, 628–637. 10.1101/gr.146985.112 23269663 PMC3613580

[B66] Ghatak A. Chaturvedi P. Weckwerth W. (2017). Cereal crop proteomics: systemic analysis of crop drought stress responses towards marker-assisted selection breeding. Front. Plant Sci. 8:757. 10.3389/fpls.2017.00757 28626463 PMC5454074

[B67] Ghatak A. Chaturvedi P. Weckwerth W. (2018). Metabolomics in plant stress physiology. Adv. Biochem. Eng. Biotechnol. 164:187–236. 10.1007/10_2017_55 29470599

[B68] Ghosh D. Xu J. (2014). Abiotic stress responses in plant roots: a proteomics perspective. Front. Plant Sci. 5:6. 10.3389/fpls.2014.00006 24478786 PMC3900766

[B69] Gjuvsland A. B. Vik J. O. Beard D. A. Hunter P. J. Omholt S. W. (2013). Bridging the genotype-phenotype gap: what does it take? J. Physiol. 591, 2055–2066. 10.1113/jphysiol.2012.248864 23401613 PMC3634519

[B70] Goh H. H. (2018). Integrative multi-omics through bioinformatics, in Omics Applications for Systems Biology. Advances in Experimental Medicine and Biology, eds Aizat W. Goh H. H. Baharum S. (Cham: Springer), 69–80. 10.1007/978-3-319-98758-3_5 30382569

[B71] Golicz A. A. Batley J. Edwards D. (2016a). Towards plant pangenomics. Plant Biotechnol. J. 14, 1099–1105. 10.1111/pbi.12499 26593040 PMC11388911

[B72] Golicz A. A. Bayer P. E. Barker G. C. Edger P. P. Kim H. Martinez P. A.. (2016b). The pangenome of an agronomically important crop plant *Brassica oleracea*. Nat. Commun. 7:13390. 10.1038/ncomms13390 27834372 PMC5114598

[B73] González R. M. Ricardi M. M. Iusem N. D. (2013). Epigenetic marks in an adaptive water stress responsive gene in tomato roots under normal and drought conditions. Epigenetics 8, 864–872. 10.4161/epi.25524 23807313 PMC3883789

[B74] Gordon S. P. Contreras-Moreira B. Woods D. P. Marais D. L. D. Burgess D. Shu S. Q.. (2017). Extensive gene content variation in the *Brachypodium distachyon* pan-genome correlates with population structure. Nat. Commun. 8:2184. 10.1038/s41467-017-02292-8 29259172 PMC5736591

[B75] Großkinsky D. K. Syaifullah S. J. Roitsch T. (2018). Integration of multi-omics techniques and physiological phenotyping within a holistic phenomics approach to study senescence in model and crop plants. J. Exp. Bot. 69, 825–844. 10.1093/jxb/erx333 29444308

[B76] Gu R. Chen F. Liu B. Wang X. Liu J. Li P.. (2015). Comprehensive phenotypic analysis and quantitative trait locus identification for grain mineral concentration, content, and yield in maize (*Zea mays* L.). Theor. Appl. Genet. 128, 1777–1789. 10.1007/s00122-015-2546-5 26058362

[B77] Gunnaiah R. Kushalappa A. C. Duggavathi R. Fox S. Somers D. J. (2012). Integrated metaboloproteomic approach to decipher the mechanisms by which wheat QTL (Fhb1) contributes to resistance against *Fusarium graminearum*. PLoS ONE 7:e40695. 10.1371/journal.pone.0040695 22866179 PMC3398977

[B78] Guo P. Baum M. Grando S. Ceccarelli S. Bai G. Li R. V. K.. (2009). Differentially expressed genes between drought-tolerant and drought-sensitive barley genotypes in response to drought stress during the reproductive stage. J. Exp. Bot. 60, 3531–3544. 10.1093/jxb/erp194 19561048 PMC2724701

[B79] Guo R. Shi L. Yan C. Zhong X. Gu F. Liu Q.. (2017). Ionomic and metabolic responses to neutral salt or alkaline salt stress in maize (*Zea mays* L.) seedlings. BMC Plant Biol. 17:41. 10.1186/s12870-017-0994-6 28187710 PMC5301417

[B80] Guo Z. Yang W. Chang Y. Ma X. Tu H. Xiong F.. (2018). Genome-wide association studies of image traits reveal genetic architecture of drought resistance in rice. Mol. Plant 11, 789–805. 10.1016/j.molp.2018.03.018 29614319

[B81] Gupta B. Saha J. Sengupta A. Gupta K. (2013). Plant abiotic stress: ‘omics’ approach. J. Plant Biochem. Physiol. 1:3. 10.4172/2329-9029.1000e10822122668

[B82] Gupta P. De B. (2017). Metabolomics analysis of rice responses to salinity stress revealed elevation of serotonin, and gentisic acid levels in leaves of tolerant varieties. Plant Signal. Behav. 12:e1335845. 10.1080/15592324 28594277 PMC5586353

[B83] Gupta P. K. Mir R. R. Mohan A. Kumar J. (2008). Wheat genomics: present status and future prospects. Int. J. Plant Genomics 36:896451. 10.1155/2008/896451 18528518 PMC2397558

[B84] He J. Zhao X. Laroche A. Lu Z. X. Liu H. Li Z. (2014). Genotyping-by-sequencing (GBS), an ultimate marker-assisted selection (MAS) tool to accelerate plant breeding. Front. Plant Sci. 5:484. 10.3389/fpls.2014.00484 25324846 PMC4179701

[B85] Henikoff S. Till B. J. Comai L. (2004). TILLING. Traditional mutagenesis meets functional genomics. Plant Physiol. 135, 630–636. 10.1104/pp.104.041061 15155876 PMC514099

[B86] Hieter P. Boguski M. (1997). Functional genomics: it's all how you read it. Science (New York, NY). 278, 601–602. 10.1126/science.278.5338.601 9381168

[B87] Hirai M.Y. Yano M. Goodenowe D. B. Kanaya S. Kimura T. Awazuhara M.. (2004). Integration of transcriptomics and metabolomics for understanding of global responses to nutritional stresses in *Arabidopsis thaliana*. Proc. Natl. Acad. Sci. U.S.A. 101, 10205–10210. 10.1073/pnas.0403218101 15199185 PMC454188

[B88] Hirsch C. N. Foerster J. M. Johnson J. M. Sekhon R. S. Muttoni G. Vaillancourt B.. (2014). Insights into the maize pan-genome and pan-transcriptome. Plant Cell 26, 121–135. 10.1105/tpc.113.119982 24488960 PMC3963563

[B89] Hittalmani S. Mahesh H. B. Shirke M. D. Biradar H. Uday G. Aruna Y. R.. (2014). Genome and Transcriptome sequence of Finger millet (*Eleusine coracana* (L.) Gaertn.) provides insights into drought tolerance and nutraceutical properties. BMC Genomics 18:465. 10.1186/s12864-017-3850-z 28619070 PMC5472924

[B90] Houle D. Govindaraju D. R. Omholt S. (2010). Phenomics: the next challenge. Nat. Rev. Genet. 11, 855–866. 10.1038/nrg2897 21085204

[B91] Huang D. Qiu Y. Zhang Y. Huang F. Meng J. Wei S.. (2013). Fine mapping and characterization of BPH27, a brown planthopper resistance gene from wild rice (*Oryza rufipogon* Griff.). Theor. Appl. Genet. 126, 219–229. 10.1007/s00122-012-1975-7 23001338

[B92] Huang L. Zhang F. Zhang F. Wang W. Zhou Y. Fu B.. (2014). Comparative transcriptome sequencing of tolerant rice introgression line and its parents in response to drought stress. BMC Genomics 15:1026. 10.1186/1471-2164-15-1026 25428615 PMC4258296

[B93] Huang X.-Y. Chao D.-Y. Gao J.-P. Zhu M.-Z. Shi M. Lin H.-X. (2009). A previously unknown zinc finger protein, DST, regulates drought and salt tolerance in rice via stomatal aperture control. Genes Dev. 23, 1805–1817. 10.1101/gad.1812409 19651988 PMC2720257

[B94] Huang X. Q. Coster H. Ganal M. W. Roder M. S. (2003). Advanced backcross QTL analysis for the identification of quantitative trait loci alleles from wild relatives of wheat (*Triticum aestivum* L.). Theor. Appl. Genet. 106, 1379–1389. 10.1007/s00122-002-1179-7 12750781

[B95] Huang X. Y. Salt D. E. (2016). Plant ionomics: from elemental profiling to environmental adaptation. Mol. Plant 9, 787–797. 10.1016/j.molp.2016.05.003 27212388

[B96] Humplík J. F. Lazár D. Husičková A. Spíchal L. (2015). Automated phenotyping of plant shoots using imaging methods for analysis of plant stress responses – a review. Plant Methods 11:29. 10.1186/s13007-015-0072-8 25904970 PMC4406171

[B97] Hurgobin B. Golicz A. A. Bayer P. E. Chan C. K. K. Tirnaz S. Dolatabadian A.. (2018). Parkin homoeologous exchange is a major cause of gene presence/absence variation in the amphidiploid *Brassica napus*. Plant Biotechnol. J. 16, 1265–1274. 10.1111/pbi.12867 29205771 PMC5999312

[B98] Jaccoud D. Peng K. Feinstein D. Kilian A. (2001). Diversity arrays: a solid state technology for sequence information independent genotyping. Nucleic Acids Res. 29:25. 10.1093/nar/29.4.e25 11160945 PMC29632

[B99] Jain S. M. Suprasanna P. (2011). Induced mutations for enhancing nutrition and food production. Gene Conserve 40, 201–215.

[B100] Jangpromma N. Songsri P. Thammasirirak S. Jaisil P. (2010). Rapid assessment of chlorophyll content in sugarcane using a spad chlorophyll meter across different water stress conditions. Asian J. Plant Sci. 9, 368–374. 10.3923/ajps.2010.368.374

[B101] Jiang W. Zhou H. Bi H. Fromm M. Yang B. (2013). Demonstration of CRISPR/Cas9/sgRNA-mediated targeted gene modification in Arabidopsis, tobacco, sorghum and rice. Nucleic Acids Res. 41:e188. 10.1093/nar/gkt780 23999092 PMC3814374

[B102] Jin Y. Yang H. Wei Z. Ma H. Ge X. (2013). Rice male development under drought stress: phenotypic changes and stage-dependent transcriptomic reprogramming. Mol. Plant 6, 1630–1645. 10.1093/mp/sst067 23604203

[B103] Jogaiah S. Govind S. R. Tran L.-S. P. (2013). Systems biology-based approaches toward understanding drought tolerance in food crops. Crit. Rev. Biotechnol. 33, 23–39. 10.3109/07388551.2012.659174 22364373

[B104] Johnson S. M. Lim F. L. Finkler A. Fromm H. Slabas A. R. Knight M. R. (2014). Transcriptomic analysis of *Sorghum bicolor* responding to combined heat and drought stress. BMC Genomics 15:456. 10.1186/1471-2164-15-456 24916767 PMC4070570

[B105] Kakumanu A. Ambavaram M. R. Klumas C. Krishnan A. Batlang U. Myers E.. (2012). Effects of drought on gene expression in maize reproductive and leaf meristem tissue revealed by RNA-seq. Plant Physiol. 160, 846–867. 10.1104/pp.112.200444 22837360 PMC3461560

[B106] Karkee M. Steward B. L. Tang L. Aziz S. A. (2009). Quantifying sub-pixel signature of paddy rice field using an artificial neural network. Comput. Electron Agric. 65, 65–76. 10.1016/j.compag.2008.07.009

[B107] Kawahara Y. Oono Y. Kanamori H. Matsumoto T. Itoh T. Minami E. (2012). Simultaneous RNA-Seq analysis of a mixed transcriptome of rice and blast fungus interaction. PLoS ONE 7:e49423. 10.1371/journal.pone.0049423 23139845 PMC3490861

[B108] Ke R. Marco M. Pacureanu A. Svedlund J. Botling J. Wählby C.. (2013). *In situ* sequencing for RNA analysis in preserved tissue and cells. Nat. Methods 10, 857–860. 10.1038/nmeth.2563 23852452

[B109] Kearsey M. J. (1998). The principles of QTL analysis (a minimal mathematics approach). J. Exp. Bot. 49, 1619–1623.

[B110] Khan F. Chai H. H. Ajmera I. Hodgman C. Mayes S. Lu C. (2017). A transcriptomic comparison of two bambara groundnut landraces under dehydration stress. Genes 8, 1–19. 10.3390/genes8040121 28420201 PMC5406868

[B111] Kikuchi J. Hirayama T. (2007). Practical aspects of uniform stable isotope labeling of higher plants for heteronuclear NMR-based metabolomics, in Metabolomics, Methods in Molecular Biology, eds Weckwerth W. (New York, NY: Humana Press), 273–286. 10.1007/978-1-59745-244-1_15 17035691

[B112] Kim H. K. Choi Y. H. Verpoorte R. (2011). NMR-based plant metabolomics: where do we stand, where do we go? Trends Biotechnol. 29, 267–275. 10.1016/j.tibtech.2011.02.001 21435731

[B113] Knoll J. E. Ramos M. L. Zeng Y. Holbrook C. C. Chow M. Chen S.. (2011). TILLING for allergen reduction and improvement of quality traits in peanut (*Arachis hypogaea* L.). BMC Plant Biol. 11:81. 10.1186/1471-2229-11-81 21569438 PMC3113929

[B114] Kover P. X. Valdar W. Trakalo J. Scarcelli N. Ehrenreich I. M. Purugganan M. D.. (2009). A multiparent advanced generation inter-cross to fine-map quantitative traits in *Arabidopsis thaliana*. PLoS Genet. 5:e1000551. 10.1371/journal.pgen.1000551 19593375 PMC2700969

[B115] Kumar A. Pathak R. K. Gupta S. M. Gaur V. S. Pandey D. (2015a). Systems biology for smart crops and agricultural innovation: filling the gaps between genotype and phenotype for complex traits linked with robust agricultural productivity and sustainability. OMICS 19, 581–601. 10.1089/omi.2015.0106 26484978 PMC4617413

[B116] Kumar A. P. K. Boualem A. Bhattacharya A. Parikh S. Desai N. Zambelli A.. (2013). SMART – sunflower mutant population and reverse genetic tools for crop improvement. BMC Plant Biol. 13:38. 10.1186/1471-2229-13-38 23496999 PMC3606330

[B117] Kumar J. Pratap A. Kumar S. (2015b). Plant phenomics: an overview, in Phenomics in Crop Plants: Trends, Options and Limitations, eds Kumar J. Pratap A. Kumar S. (New Delhi: Springer), 1–10. 10.1007/978-81-322-2226-2_1

[B118] Kumari A. Das P. Parida A. K. Agarwal P. K. (2015). Proteomics, metabolomics, and ionomics perspectives of salinity tolerance in halophyte. Front. Plan Sci. 6:537. 10.3389/fpls.2015.00537 26284080 PMC4518276

[B119] Kuo T. C. Tian T. F. Tseng Y. J. (2013). 3Omics: a web-based systems biology tool for analysis, integration and visualization of human transcriptomic, proteomic and metabolomic data. BMC Syst. Biol. 7:64. 10.1186/1752-0509-7-64 23875761 PMC3723580

[B120] Kurowska M. Daszkowska-Golec A. Gruszka D. Marzec M. Szurman M. Szarejko I.. (2011). TILLING: a shortcut in functional genomics. J. Appl. Genet. 52, 371–390. 10.1007/s13353-011-0061-1 21912935 PMC3189332

[B121] Laloum T. Martín G. Duque P. (2018). Alternative splicing control of abiotic stress responses. Trends Plant Sci. 23, 140–150. 10.1016/j.tplants.2017.09.019 29074233

[B122] Larrainzar E. Wienkoop S. Scherling C. Kempa S. Ladrera R. Arrese-Igor C.. (2009). Carbon metabolism and bacteroid functioning are involved in the regulation of nitrogen fixation in *Medicago truncatula* under drought and recovery. Mol. Plant Microbe Interact. 22, 1565–1576. 10.1094/MPMI-22-12-1565 19888822

[B123] Larrainzar E. Wienkoop S. Weckwerth W Ladrera R. Arrese-Igor C. González E.M. (2007). *Medicago truncatula* root nodule proteome analysis reveals differential plant and bacteroid responses to drought stress. Plant Physiol. 144, 1495–1507. 10.1104/pp.107.101618 17545507 PMC1914115

[B124] Lasky J. R. Upadhyaya H. D. Ramu P. Deshpande S. Hash C. T. Bonnette J.. (2015). Genome-environment associations in sorghum landraces predict adaptive traits. Sci. Adv. 1:e1400218. 10.1126/sciadv.1400218 26601206 PMC4646766

[B125] Lawrenson T. Shorinola O. Stacey N. Li C. Ostergaard L. Patron N.. (2015). Induction of targeted, heritable mutations in barley and *Brassica oleracea* using RNA-guided Cas9 nuclease. Genome Biol. 16:258. 10.1186/s13059-015-0826-7 26616834 PMC4663725

[B126] Le D. T. Nishiyama R. Watanabe Y. Tanaka M. Seki M. Le H.. (2012). Differential gene expression in soybean leaf tissues at late developmental stages under drought stress revealed by genome-wide transcriptome analysis. PLoS ONE 7:e49522. 10.1371/journal.pone.0049522 23189148 PMC3505142

[B127] Le G. H. Fontaine J. X. Molinie R. Pelloux J. Mesnard F. Gillet F.. (2017). NMR-based metabolomics to study the cold-acclimation strategy of two *Miscanthus* genotypes. Phytochem. Anal. 28, 58–67. 10.1002/pca.2649 27976469

[B128] Li G. Y. Zhou J. X. Ma W. K. Jiang L. G. Jin Z. H. Zhang Y.. (2014). *De novo* assembly of soybean wild relatives for pan-genome analysis of diversity and agronomic traits. Nat. Biotechnol. 32, 1045–1052. 10.1038/nbt.2979 25218520

[B129] Li Y. Zou W. Lin S. Onofua D. Yang Z. Chen H.. (2017). Transcriptome profiling and digital gene expression analysis of sweet potato for the identification of putative genes involved in the defense response *against Fusarium oxysporum* f. sp. batatas. PLoS ONE 12:e0187838. 10.1371/journal.pone.0187838 29131830 PMC5683638

[B130] Li Y. F. Wang Y. I. Tang Y. Kakani V. G. Mahalingam R. (2013). Transcriptome analysis of heat stress response in switchgrass (*Panicum virgatum* L.). BMC Plant Biol. 13:153. 10.1186/1471-2229-13-153 24093800 PMC3851271

[B131] Li Z. Liu Z. B. Xing A. Moon B. P. Koellhoffer J. P. Huang L.. (2015). Cas9-guide RNA directed genome editing in soybean. Plant Physiol. 169, 960–970. 10.1104/pp.15.00783 26294043 PMC4587461

[B132] Lin K. Zhang N. Severing E. I. Nijveen H. Cheng F. Visser R. G.. (2014). Beyond genomic variation-comparison and functional annotation of three *Brassica rapa* genomes: a turnip, a rapid cycling and a Chinese cabbage. BMC Genomics 15:250. 10.1186/1471-2164-15-250 24684742 PMC4230417

[B133] Lindon J. C. Nicholson J. K. (2008). Analytical technologies for metabonomics and metabolomics, and multi-omic information recovery. Trends Anal. Chem. 27, 194–204. 10.1016/j.trac.2007.08.009

[B134] Liu B. Zhang N. Zhao S. Chang J. Wang Z. Zhang G.. (2015). Proteomic changes during tuber dormancy release process revealed by iTRAQ quantitative proteomics in potato. Plant Physiol. Biochem. 86, 181–190. 10.1016/j.plaphy.2014.12.003 25514565

[B135] Liu D. Ford K. L. Roessner U. Natera S. Cassin A. M. Patterson J. H.. (2013). Rice suspension cultured cells are evaluated as a model system to study salt responsive networks in plants using a combined proteomic and metabolomic profiling approach. Proteomics 13, 2046–2206. 10.1002/pmic.201200425 23661342

[B136] Lu F. Romay M. C. Glaubitz J. C. Bradbury P. J. Elshire R. J. Wang T.. (2015). High-resolution genetic mapping of maize pan-genome sequence anchors. Nat. Commun. 6:6914. 10.1038/ncomms7914 25881062 PMC4411285

[B137] Lu X. Wang X. Chen X. Shu N. Wang J. Wang D.. (2017). Single-base resolution methylomes of upland cotton (*Gossypium hirsutum* L.) reveal epigenome modifications in response to drought stress. BMC Genomics 18:297. 10.1186/s12864-017-3681-y 28407801 PMC5390369

[B138] Lueong S. S. Hoheisel J. D. Alhamdani M. S. S. (2014). Protein microarrays as tools for functional proteomics: achievements, promises and challenges. J. Proteomics Bioinform. 7:1–10. 10.4172/jpb.S7-004

[B139] Luo J. (2015). Metabolite-based genome-wide association studies in plants. Curr. Opin. Plant Biol. 24, 31–38. 10.1016/j.pbi.2015.01.006 25637954

[B140] Mace E. S. Tai S. Gilding E. K. Li Y. Prentis P. J. Bian L.. (2013). Whole-genome sequencing reveals untapped genetic potential in Africa's indigenous cereal crop sorghum. Nat. Commun. 4:2320. 10.1038/ncomms3320 23982223 PMC3759062

[B141] Magalhaes J. V. Liu J. Guimaraes C. T. Lana U. G. P. Alves V. M. C. Wang Y. H.. (2007). A gene in the multidrug and toxic compound extrusion (MATE) family confers aluminum tolerance in sorghum. Nat. Genet. 39, 1156–1161. 10.1038/ng2074 17721535

[B142] Mahlein A. K. (2016). Plant disease detection by imaging sensors–parallels and specific demands for precision agriculture and plant phenotyping. Plant Dis. 100, 241–251. 10.1094/PDIS-03-15-0340-FE 30694129

[B143] Maksup S. Roytrakul S. Supaibulwatana K. (2014). Physiological and comparative proteomic analyses of Thai jasmine rice and two check cultivars in response to drought stress. J. Plant Interact. 9, 43–55. 10.1080/17429145.2012.752042

[B144] Mangin B. Casadebaig P. Cadic E. Blanchet N. Boniface M. C. Carrère S.. (2017). Genetic control of plasticity of oil yield for combined abiotic stresses using a joint approach of crop modelling and genome-wide association. Plant Cell Environ. 40, 2276–2291. 10.1111/pce.12961 28418069

[B145] Margaria P. Abbà Palmano S. S. (2013). Novel aspects of grapevine response to phytoplasma infection investigated by a proteomic and phosphoproteomic approach with data integration into functional networks. BMC Genomics 14:38. 10.1186/1471-2164-14-38 23327683 PMC3564869

[B146] Mascher M. Gerlach N. Gahrtz M. Bucher M. Scholz U. Dresselhaus T. (2014). Sequence and ionomic analysis of divergent strains of maize inbred line B73 with an altered growth phenotype. PLoS ONE 9:e96782. 10.1371/journal.pone.0096782 24804793 PMC4013074

[B147] Massa A. N. Childs K. L. Buell C. R. (2013). Abiotic and biotic stress responses in *Solanum tuberosum* group phureja DM1-3516R44 as measured through whole transcriptome sequencing. Plant Gen. 6:15. 10.3835/plantgenome2013.05.0014

[B148] Matsuda F. Nakabayashi R. Yang Z. Okazaki Y. Yonemaru J. Ebana K.. (2015). Metabolome-genome-wide association study dissects genetic architecture for generating natural variation in rice secondary metabolism. Plant J. 81, 13–23. 10.1111/tpj.12681 25267402 PMC4309412

[B149] Matsui A. Ishida J. Morosawa T. Mochizuki Y. Kaminuma E. Endo T. A.. (2008). *Arabidopsis* transcriptome analysis under drought, cold, high-salinity and ABA treatment conditions using a tiling array. Plant Cell Physiol. 49, 1135–1149. 10.1093/pcp/pcn101 18625610

[B150] Mba C. (2013). Induced mutations unleash the potentials of plant genetic resources for food and agriculture. Agronomy 3, 200–231. 10.3390/agronomy3010200

[B151] McCallum C. M. Comai L. Greene E. A. Henikoff S. (2000). Targeting induced local lesions IN genomes (TILLING) for plant functional genomics. Plant Physiol. 123, 439–442. 10.1104/pp.123.2.439 10859174 PMC1539256

[B152] McGrail R. K. Van Sanford D. A. McNear D. H. Jr. (2020). Trait-based root phenotyping as a necessary tool for crop selection and improvement. Agronomy 10:1328. 10.3390/agronomy10091328

[B153] McLuckey S. A. Stephenson J. L. Jr. (1998). Ion/ion chemistry of high-mass multiply charged ions. Mass Spectrom. Rev. 17, 369–407. 10.1002/(SICI)1098-2787(1998)17:6<369::AID-MAS1>3.0.CO;2-J 10360331

[B154] Millet E. J. Welcker C. Kruijer W. Negro S. Coupel-Ledru A. Nicolas S. D.. (2016). Genome-wide analysis of yield in Europe: allelic effects vary with drought and heat scenarios. Plant Physiol. 172, 749–764. 10.1104/pp.16.00621 27436830 PMC5047082

[B155] Minoia S. Petrozza A. D'Onofrio O. Piron F. Mosca G. Sozio G.. (2010). A new mutant genetic resource for tomato crop improvement by TILLING technology. BMC Res. Not. 3:69. 10.1186/1756-0500-3-69 20222995 PMC2845601

[B156] Mittova V. Guy M. Tal M. Volokita M. (2004). Salinity up-regulates the antioxidative system in root mitochondria and peroxisomes of the wild salt-tolerant tomato species lycopersicon. J. Exp. Bot. 55, 1105–1113. 10.1093/jxb/erh113 15047761

[B157] Moco S. Bino R. J. De Vos R. C. H. Vervoort J. (2007). Metabolomics technologies and metabolite identification. Trends Analyt. Chem. 26, 855–866. 10.1016/j.trac.2007.08.003

[B158] Mohammadi P. P. Moieni A. Komatsu S. (2012). Comparative proteome analysis of drought-sensitive and drought-tolerant rapeseed roots and their hybrid F1 line under drought stress. Amino Acids 43, 2137–2152. 10.1007/s00726-012-1299-6 22543724

[B159] Montenegro J. D. Golicz A. A. Bayer P. E. Hurgobin B. Lee H. Chan C. K. K.. (2017). The pangenome of hexaploid bread wheat. Plant J. 90, 1007–1013. 10.1111/tpj.13515 28231383

[B160] Mosa K. A. Ismail A. Helmy M. (2017). Omics and system biology approaches in plant stress research, in Plant Stress Tolerance: an Integrated Omics Approach, eds Mosa K. A. Ismail A. Helmy M. (Cham: Springer), 21–34. 10.1007/978-3-319-59379-1_2

[B161] Muthamilarasan M. Prasad M. (2017). Genetic determinants of drought stress tolerance in Setaria, in Genetics and Genomics of Setaria. Plant Genetics and Genomics: Crops and Models, Vol. 19, eds Doust A. Diao X. (Cham: Springer) 267–289. 10.1007/978-3-319-45105-3_16

[B162] Muthamilarasan M. Singh N. K. Prasad M. (2019). Multi-omics approaches for strategic improvement of stress tolerance in underutilized crop species: a climate change perspective. Adv. Genet. 103, 1–38. 10.1016/bs.adgen.2019.01.001 30904092

[B163] Muthurajan R. Shobbar Z. S. Jagadish S. Bruskiewich R. Ismail A. Leung H.. (2011). Physiological and proteomic responses of rice peduncles to drought stress. Mol. Biotechnol. 48, 173–182. 10.1007/s12033-010-9358-2 21132544

[B164] Nakagami H. Sugiyama N. Ishihama Y. Shirasu K. (2012). Shotguns in the front line: phosphoproteomics in plants. Plant Cell Physiol. 53, 118–124. 10.1093/pcp/pcr148 22039104

[B165] Nataraja K. N. Madhura B. G. Parvathi S. M. (2017). Omics: modern tools for precise understanding of drought adaptation in plants, in Plant OMICS and Crop Breeding, eds Zargar S. M. Rai V. (Palm Bay, FL: Apple Academic Press), 289–320.

[B166] Nekrasov V. Wang C. Win J. Lanz C. Weigel D. Kamoun S. (2017). Rapid generation of a transgene-free powdery mildew resistant tomato by genome deletion. Sci. Rep. 7:482. 10.1038/s41598-017-00578-x 28352080 PMC5428673

[B167] Neveu P. Tireau A. Hilgert N. Nègre V. Mineau-Cesari J. Brichet N.. (2019). Dealing with multi-source and multi-scale information in plant phenomics: the ontology-driven phenotyping hybrid information system. New Phytol. 221, 588–601. 10.1111/nph.15385 30152011 PMC6585972

[B168] Norton G. J. Deacon C. M. Xiong L. Z. Huang S. Y. Meharg A. A. Price A. H. (2010). Genetic mapping of the rice ionome in leaves and grain: identification of QTLs for 17 elements including arsenic, cadmium, iron and selenium. Plant Soil 329, 139–153. 10.1007/s11104-009-0141-8

[B169] Nouri M. Z. Komatsu S. (2010). Comparative analysis of soybean plasma membrane proteins under osmotic stress using gel-based and LC MS/MS-based proteomics approaches. Proteomics 10, 1930–1945. 10.1002/pmic.200900632 20209511

[B170] Novik K. L. Nimmrich I. Genc B. Maier S. Piepenbrock C. Olek A.. (2002). Epigenomics: genome-wide study of methylation phenomena. Curr. Issues Mol. Biol. 4, 111–128. 12432963

[B171] Offermann S. Danker T. Dreymuller D. Kalamajka R. Topsch S. Weyand K.. (2006). Illumination is necessary and sufficient to induce histone acetylation independent of transcriptional activity at the C4-specific phosphoenolpyruvate carboxylase promoter in maize. Plant Physiol. 141, 1078–1088. 10.1104/pp.106.080457 16679423 PMC1489893

[B172] Okay S. Derelli E. Unver T. (2014). Transcriptome-wide identification of bread wheat WRKY transcription factors in response to drought stress. Mol. Genet. Genomics 289, 765–781. 10.1007/s00438-014-0849-x 24748053

[B173] Ong-Abdullah M. Ordway J. M. Jiang N. Ooi S. E. Kok S. Y. Sarpan N.. (2015). Loss of karma transposon methylation underlies the mantled somaclonal variant of oil palm. Nature 525, 533–537. 10.1038/nature15365 26352475 PMC4857894

[B174] Paine J. A. Shipton C. A. Chaggar S. Howells R. M. Kennedy M. J. Vernon G.. (2005). Improving the nutritional value of Golden Rice through increased pro-vitamin A content. Nat. Biotechnol. 23, 482–487. 10.1038/nbt1082 15793573

[B175] Pandey G. Yadav C. B. Sahu P. P. Muthamilarasan M. Prasad M. (2017). Salinity induced differential methylation patterns in contrasting cultivars of foxtail millet (*Setaria italica* L.). Plant Cell Rep. 36, 759–772. 10.1007/s00299-016-2093-9 27999979

[B176] Pandey M. K. Roorkiwal M. Singh V. K. Ramalingam A. Kudapa H. Thudi M.. (2016). Emerging genomic tools for legume breeding: current status and future perspectives. Front. Plant Sci. 7:455. 10.3389/fpls.2016.00455 27199998 PMC4852475

[B177] Parent S. E. Parent L. E. Egozcue J. J. Rozane D. E. Hernandes A. Lapointe L.. (2013). The plant ionome revisited by the nutrient balance concept. Front. Plant Sci. 4:39. 10.3389/fpls.2013.00039 23526060 PMC3605521

[B178] Parker D. Beckmann M. Zubair H. Enot D. P. Caracuel-Rios Z. Overy D. P.. (2009). Metabolomic analysis reveals a common pattern of metabolic re-programming during invasion of three host plant species by *Magnaporthe grisea*. Plant J. 59, 723–737. 10.1111/j.1365-313X.2009.03912.x 19453445

[B179] Pathan M. S. Sleper D. A. (2008). Advances in soybean breeding, in Genetics and Genomics of Soybean, Vol. 2, ed Stacey G. (New York, NY: Springer), 113–133. 10.1007/978-0-387-72299-3_8

[B180] Paupiere M. J. Muller F. Li H. J. Rieu I. Tikunov Y. M. Visser R. G. F.. (2017). Untargeted metabolomic analysis of tomato pollen development and heat stress response. Plant Reprod. 30, 81–94. 10.1007/s00497-017-0301-6 28508929 PMC5486769

[B181] Penna S. Jain S. M. (2017). Mutant resources and mutagenomics in crop plants. Emirates J. Food Agric. 29, 651–657. 10.9755/ejfa.2017.v29.i9.86

[B182] Pieruschka R. Klimov D. Kolber Z. Berry J. A. (2010). Monitoring of cold and light stress impact on photosynthesis by using the laser induced fluorescence transient (LIFT) approach. Funct. Plant Biol. 37, 395–402. 10.1071/FP09266

[B183] Pinson S. R. M. Tarpley L. Yan W. Yeater K. Lahner B. Yakubova E.. (2015). Worldwide genetic diversity for mineral element concentrations in rice grain. Crop Sci. 55, 294–311. 10.2135/cropsci2013.10.0656

[B184] Pinu F. R. Beale D. J. Paten A. M. Kouremenos K. Swarup S. Wishart D.. (2019). Systems biology and multi-omics integration: viewpoints from the metabolomics research community. Metabolites 9:76. 10.3390/metabo9040076 31003499 PMC6523452

[B185] Qi X. Xie S. Liu Y. Yi F. Yu J. (2013). Genome-wide annotation of genes and noncoding RNAs of foxtail millet in response to simulated drought stress by deep sequencing. Plant Mol. Biol. 83, 459–473. 10.1007/s11103-013-0104-6 23860794

[B186] Rabello A. R. Guimarães C. M. Rangel P. H. da Silva F. R. Seixas D. de Souza E.. (2008). Identification of drought-responsive genes in roots of upland rice (*Oryza sativa* L.). BMC Genomics 9:485. 10.1186/1471-2164-9-485 18922162 PMC2605477

[B187] Rabouam C. Comes A. M. Bretagnolle V. Humbert J. F. Periquet G. Bigot Y. (1999). Features of DNA fragments obtained by random amplified polymorphic DNA (RAPD) assays. Mol Ecol. 8, 493–503. 10.1046/j.1365-294X.1999.00605.x 10199010

[B188] Ram T. Majumder N. D. Krishnaveni D. Ansari M. M. (2007). Rice variety Dhanrasi, an example of improving yield potential and disease resistance by introgressing gene(s) from wild species (*Oryza rufipogon*). Curr. Sci. 92, 987–992.

[B189] Ramalingam A. Kudapa H. Pazhamala L. T. Weckwerth W. Varshney R. K. (2015). Proteomics and metabolomics: two emerging areas for legume improvement. Front. Plant Sci. 6:1116. 10.3389/fpls.2015.01116 26734026 PMC4689856

[B190] Rascher U. Pieruschka R. (2008). Spatio-temporal variations of photosynthesis: the potential of optical remote sensing to better understand and scale light use efficiency and stresses of plant ecosystems. Precis. Agric. 9, 355–366. 10.1007/s11119-008-9074-0

[B191] Raza A. Tabassum J. Kudapa H. Varshney R. K. (2021). Can omics deliver temperature resilient ready-to-grow crops? Crit. Rev. Biotechnol. 2021, 1–24. 10.1080/07388551.2021.1898332 33827346

[B192] Reynolds D. Ball J. Bauer A. Davey R. Griffiths S. Zhou J. (2019). CropSight: a scalable and open-source information management system for distributed plant phenotyping and IoT-based crop management. Gigascience 8, 1–35. 10.1093/gigascience/giz009 30715329 PMC6423370

[B193] Rinaldo A. R. Ayliffe M. (2015). Gene targeting and editing in crop plants: a new era of precision opportunities. Mol. Breed. 35:40. 10.1007/s11032-015-0210-z

[B194] Ritchie M. D. Holzinger E. R. Li R. W. Pendergrass S. A. Kim D. (2015). Methods of integrating data to uncover genotype-phenotype interactions. Nat. Rev. Genet. 16, 85–97. 10.1038/nrg3868 25582081

[B195] Roitsch T. Cabrera-Bosquet L. Fournier A. Ghamkhar K. Jiménez-Berni J. Pinto F.. (2019). Review: new sensors and data-driven approaches—a path to next generation phenomics. Plant Sci. 282, 2–10. 10.1016/j.plantsci.2019.01.011 31003608 PMC6483971

[B196] Ruiz-Garcia L. Lunadei L. Barreiro P. Robla J. I. (2009). A review of wireless sensor technologies and applications in agriculture and food industry: state of the art and current trends. Sensors 9, 4728–4750. 10.3390/s90604728 22408551 PMC3291936

[B197] Saand M. A. Xu Y. P. Li W. Wang J. P. Cai X. Z. (2015). Cyclic nucleotide gated channel gene family in tomato: genome-wide identification and functional analyses in disease resistance. Front. Plant Sci. 6:303. 10.3389/fpls.2015.00303 25999969 PMC4419669

[B198] Saito K. Matsuda F. (2010). Metabolomics for functional genomics, systems biology, and biotechnology. Annu. Rev. Plant Biol. 61, 463–448. 10.1146/annurev.arplant.043008.092035 19152489

[B199] Salekdeh G. H. Siopongco J. Wade L. J. Ghareyazie B. Bennett J. (2002). Proteomic analysis of rice leaves during drought stress and recovery. Proteomics 2, 1131–1145. 10.1002/1615-9861(200209)2:9<1131::AID-PROT1131>3.0.CO 12362332

[B200] Sali A. Glaeser R. Earnest T. Baumeister W. (2003). From words to literature in structural proteomics. Nature 422, 216–225. 10.1038/nature01513 12634795

[B201] Salt D. E. Baxter I. Lahner B. (2008). Ionomics and the study of the plant ionome. Annu. Rev. Plant Biol. 59, 709–733. 10.1146/annurev.arplant.59.032607.092942 18251712

[B202] Sana T. R. Fischer S. Wohlgemuth G. Katrekar A. Jung K. H. Ronald P. C.. (2010). Metabolomic and transcriptomic analysis of the rice response to the bacterial blight pathogen *Xanthomonas oryzae* pv. oryzae. Metabolomics 6, 451–465. 10.1007/s11306-010-0218-7 20676379 PMC2899020

[B203] Sanchez-Rodríguez E. Md M. Rubio-Wilhelmi Cervilla L. M. Blasco B. Rios J. J.. (2010). Study of the ionome and uptake fluxes in cherry tomato plants under moderate water stress conditions. Plant Soil 335, 339–347. 10.1007/s11104-010-0422-2

[B204] Saranga Y. Jiang C. X. Wright R. J. Yakir D. Paterson A. H. (2004). Genetic dissection of cotton physiological responses to arid conditions and their inter-relationships with productivity. Plant Cell Environ. 27, 263–277. 10.1111/j.1365-3040.2003.01134.x

[B205] Satismruti K. Senthil N. Vellaikumar S. Ranjani R. V. Raveendran M. (2013). Plant Ionomics: a platform for identifying novel gene regulating plant mineral nutrition. Am. J. Plant Sci. 4, 1309–1315. 10.4236/ajps.2013.47162

[B206] Sauter H. Lauer M. Fritsch H. (1991). Metabolic profiling of plants – a new diagnostic technique”, in Synthesis and Chemistry of Agrochemicals II, eds Baker D. R. Fenyes J. G. Moberg W. K. (Washington, DC: American Chemical Society), 288–299. 10.1021/bk-1991-0443.ch024

[B207] Schatz M. C. Maron L. G. Stein J. C. Wences A. H. Gurtowski J. Biggers E.. (2014). Whole genome *de novo* assemblies of three divergent strains of rice, *Oryza sativa*, document novel gene space of aus and indica. Genome Biol. 15:506. 10.1186/PREACCEPT-2784872521277375 25468217 PMC4268812

[B208] Schmitz R. J. He Y. Valdés-López O. Khan S. M. Joshi T. Urich M. A.. (2013). Epigenome-wide inheritance of cytosine methylation variants in a recombinant inbred population. Genome Res. 23, 1663–1674. 10.1101/gr.152538.112 23739894 PMC3787263

[B209] Schnurbusch T. Hayes J. Sutton T. (2010). Boron toxicity tolerance in wheat and barley: Australian perspectives. Breed. Sci. 60, 297–304. 10.1270/jsbbs.60.297

[B210] Semel Y. Schauer N. Roessner U. Zamir D. Fernie A. R. (2007). Metabolite analysis for the comparison of irrigated and non-irrigated field grown tomato of varying genotype. Metabolomics 3, 289–295. 10.1007/s11306-007-0055-5

[B211] Shah T. Xu J. Zou X. Cheng Y. Nasir M. Zhang X. (2018). Omics approaches for engineering wheat production under abiotic stresses. Int. J. Mol. Sci. 19, 2390–2405. 10.3390/ijms19082390 30110906 PMC6121627

[B212] Shalata A. Mittova V. Volokita M. Guy M. Tal M. (2001). Response of the cultivated tomato and its wild salt-tolerant relative *Lycopersicon pennellii* to salt-dependent oxidative stress: the root antioxidative system. Physiol. Plant. 112, 487–494. 10.1034/j.1399-3054.2001.1120405.x 11473708

[B213] Shikha M. Kanika A. Rao A. R. Mallikarjuna M. G. Gupta H. S. Nepolean T. (2017). Genomic selection for drought tolerance using genome-wide SNPs in maize. Front. Plant Sci. 8:550. 10.3389/fpls.2017.00550 28484471 PMC5399777

[B214] Silvente S. Sobolev A. P. Lara M. (2012). Metabolite adjustments in drought tolerant and sensitive soybean genotypes in response to water stress. PLoS ONE 7:e38554. 10.1371/journal.pone.0038554 22685583 PMC3369847

[B215] Singh U. M. Sareen P. Sengar R. S. Kumar A. (2013). Plant ionomics: a newer approach to study mineral transport and its regulation. Acta Physiol. Plant 35, 2641–2653. 10.1007/s11738-013-1316-8

[B216] Sirault X. R. R. James R. A. Furbank R. T. (2009). A new screening method for osmotic component of salinity tolerance in cereals using infrared thermography. Funct. Plant Biol. 36, 970–977. 10.1071/FP09182 32688708

[B217] Spindel J. E. Dahlberg J. Colgan M. Hollingsworth J. Sievert J. Staggenborg S. H.. (2018). Association mapping by aerial drone reveals 213 genetic associations for *Sorghum bicolor* biomass traits under drought. BMC Genomics 19:679. 10.1186/s12864-018-5055-5 30223789 PMC6142696

[B218] Strahl B. Allis C. (2000). The language of covalent histone modifications. Nature 403, 41–45. 10.1038/47412 10638745

[B219] Stroud H. Ding B. Simon S. A. Feng S. Bellizzi M. Pellegrini M.. (2013). Plants regenerated from tissue culture contain stable epigenome changes in rice. Elife 2:e00354. 10.7554/eLife.00354 23539454 PMC3601819

[B220] Subba P. Kumar R. Gayali S. Shekhar S. Parveen S. Pandey A.. (2013). Characterisation of the nuclear proteome of a dehydration-sensitive cultivar of chickpea and comparative proteomic analysis with a tolerant cultivar. Proteomics 13, 1973–1992. 10.1002/pmic.201200380 23798506

[B221] Sumner L. W. Mendes P. Dixon R. A. (2003). Plant metabolomics: large-scale phytochemistry in the functional genomics era. Phytochemistry 62, 817–836. 10.1016/s0031-9422(02)00708-2 12590110

[B222] Sun C. X. Gao X. X. Li M. Q. Fu J. Q. Zhang Y. L. (2016). Plastic responses in the metabolome and functional traits of maize plants to temperature variations. Plant Biol. 18, 249–261. 10.1111/plb.12378 26280133

[B223] Sun X. Weckwerth W. (2012). COVAIN: a toolbox for uni- and multivariate statistics, time-series and correlation network analysis and inverse estimation of the differential Jacobian from metabolomics covariance data. Metabolomics 8, 81–93. 10.1007/s11306-012-0399-3

[B224] Suzuki T. Eiguchi M. Kumamaru T. Satoh H. Matsusaka H. Moriguchi K.. (2008). MNU-induced mutant pools and high performance TILLING enable finding of any gene mutation in rice. Mol. Genet. Genomics 279, 213–223. 10.1007/s00438-007-0293-2 17952471

[B225] Svitashev S. Young J. K. Schwartz C. Gao H. Falco S. C. Cigan A. M. (2015). Targeted mutagenesis, precise gene editing, and site-specific gene insertion in maize using Cas9 and guide RNA. Plant Physiol. 169, 931–945. 10.1104/pp.15.00793 26269544 PMC4587463

[B226] Swarbrick P. J. Schulze-Lefert P. Scholes J. D. (2006). The metabolic consequences of susceptibility and the activation of race specific or broad spectrum resistance pathways in barley leaves challenged with the powdery mildew fungus. Plant Cell Environ. 29, 1061–1076. 10.1111/j.1365-3040.2005.01472.x 17080933

[B227] Talukdar D. Sinjushin A. (2015). Cytogenomics and mutagenomics in plant functional biology and breeding, in PlantOmics: The Omics of Plant Science, eds Barh D. Khan M. Davies E. (New Delhi: Springer), 113–156. 10.1007/978-81-322-2172-2_5

[B228] Tanaka K. Waki H. Ido Y. Akita S. Yoshida Y. Yoshida T. (1988). Protein and polymer analyses up to m/z 100000 by laser ionization time-of-flight mass spectrometry. Rapid Commun. Mass Spectrom. 2, 151–153. 10.1002/rcm.1290020802

[B229] Tao Y. Mace E. George-Jaeggli B. Hunt C. Cruickshank A. Henzell R.. (2018). Novel grain weight loci revealed in a cross between cultivated and wild sorghum. Plant Genome 11, 1–10. 10.3835/plantgenome2017.10.0089 30025022 PMC12810130

[B230] Tao Y. Mace E. S. Tai S. Cruickshank A. Campbell B. C. Zhao X.. (2017). Whole-genome analysis of candidate genes associated with seed size and weight in *sorghum bicolor* reveals signatures of artificial selection and insights into parallel domestication in cereal crops. Front. Plant Sci. 8:1237. 10.3389/fpls.2017.01237 28769949 PMC5513986

[B231] Tettelin H. Masignani V. Cieslewicz M. J. Donati C. Medini D. Ward N. L.. (2005). Genome analysis of multiple pathogenic isolates of Streptococcus agalactiae: implications for the microbial 'pan-genome'. Proc. Natl. Acad. Sci. U.S.A. 102, 13950–13955. 10.1073/pnas.0506758102 16172379 PMC1216834

[B232] Till B. J. Reynolds S. H. Weil C. Springer N. Burtner C. Young K.. (2004). Discovery of induced point mutations in maize genes by TILLING. BMC Plant Biol. 4:12. 10.1186/1471-2229-4-12 15282033 PMC512284

[B233] Todaka D. Shinozaki K. Yamaguchi-Shinozaki K. (2015). Recent advances in the dissection of drought-stress regulatory networks and strategies for development of drought-tolerant transgenic rice plants. Front. Plant Sci. 6:84. 10.3389/fpls.2015.00084 25741357 PMC4332304

[B234] Tokimatsu T. Sakurai N. Suzuki H. Ohta H. Nishitani K. Koyama T.. (2005). KaPPA-view: a web-based analysis tool for integration of transcript and metabolite data on plant metabolic pathway maps. Plant Physiol. 138, 1289–1300. 10.1104/pp.105.060525 16010003 PMC1176402

[B235] Tomlekova N. B. (2010). Induced mutagenesis for crop improvement in Bulgaria. Plant Mutat. Rep. 2, 4–27.

[B236] Toorchi M. Yukawa K. Nouri M. Z. Komatsu S. (2009). Proteomics approach for identifying osmotic-stress-related proteins in soybean roots. Peptides 30, 2108–2117. 10.1016/j.peptides.2009.09.006 19747515

[B237] Twyman R. M. (2013). Principles of Proteomics. Abingdon: Garland Science Press.

[B238] Urano K. Kurihara Y. Seki M. Shinozaki K. (2010). “Omics” analyses of regulatory networks in plant abiotic stress responses. Curr. Opin. Plant Biol. 13, 132–113. 10.1016/j.pbi.2009.12.006 20080055

[B239] Urbanczyk-Wochniak E. Luedemann A. Kopka J. Selbig J. Roessner-Tunali U. Willmitzer L.. (2003). Parallel analysis of transcript and metabolic profiles: a new approach in systems biology. EMBO Rep. 4, 989–993. 10.1038/sj.embor.embor944 12973302 PMC1326402

[B240] van Dijk K. Ding Y. Malkaram S. Riethoven J. J. M. Liu R. Yang J.. (2010). Dynamic changes in genome-wide histone H3 lysine 4 methylation patterns in response to dehydration stress in *Arabidopsis thaliana*. BMC Plant Biol. 10:238. 10.1186/1471-2229-10-238 21050490 PMC3095321

[B241] Varshney R. K. Thudi M. May G. D. Jackson S. A. (2010). Legume genomics and breeding. Plant Breed. Rev. 33, 257–304. 10.1002/9780470535486.ch6

[B242] Viant M. R. Sommer U. (2013). Mass spectrometry based environmental metabolomics: a primer and review. Metabolomics 9, 144–158. 10.1007/s11306-012-0412-x

[B243] Vos P. Hogers R. Bleeker M. Reijans M. Lee T. Hornes M.. (1995). AFLP: a new technique for DNA fingerprinting. Nucleic Acids Res. 23, 4407–4414. 10.1093/nar/23.21.4407 7501463 PMC307397

[B244] Wang M. Wang P. Tu L. Zhu S. Zhang L. Li Z.. (2016). Multi-omics maps of cotton fibre reveal epigenetic basis for staged single-cell differentiation. Nucleic Acids Res. 44, 4067–4079. 10.1093/nar/gkw238 27067544 PMC4872108

[B245] Wang W. S. Mauleon R. Hu Z. Q. Chebotarov D. Tai S. S. Wu Z. C.. (2018). Genomic variation in 3,010 diverse accessions of Asian cultivated rice. Nature 557, 43–49. 10.1038/s41586-018-0063-9 29695866 PMC6784863

[B246] Wang Y. Cheng X. Shan Q. Zhang Y. Liu J. Gao C.. (2014). Simultaneous editing of three homoeoalleles in hexaploid bread wheat confers heritable resistance to powdery mildew. Nat. Biotechnol. 32, 947–951. 10.1038/nbt.2969 25038773

[B247] Weckwerth W. (2003). Metabolomics in systems biology. Annu. Rev. Plant Biol. 54, 669–689. 10.1146/annurev.arplant.54.031902.135014 14503007

[B248] Weckwerth W. (2010). Metabolomics: an integral technique in systems biology. Bioanalysis 2, 829–836. 10.4155/bio.09.192 21083277

[B249] Weckwerth W. (2011a). Green systems biology – from single genomes, proteomes and metabolomes to ecosystems research and biotechnology. J. Proteome 75, 284–305. 10.1016/j.jprot.2011.07.010 21802534

[B250] Weckwerth W. (2011b). Unpredictability of metabolism—the key role of metabolomics science in combination with next-generation genome sequencing. Anal. Bioanal. Chem. 400:1967. 10.1007/s00216-011-4948-9 21556754 PMC3098350

[B251] Weckwerth W. (2019). Toward a unification of system-theoretical principles in biology and ecology—the stochastic lyapunov matrix equation and its inverse application. Front. Appl. Math. Stat. 5:29. 10.3389/fams.2019.00029

[B252] Weckwerth W. Fiehn O. (2002). Can we discover novel pathways using metabolomic analysis? Curr. Opin. Biotechnol. 13, 156–160. 10.1016/s0958-1669(02)00299-9 11950569

[B253] Weckwerth W. Ghatak A. Bellaire A. Chaturvedi P. Varshney R. K. (2020). PANOMICS meets germplasm. Plant Biotechnol. J. 10:1111. 10.1111/pbi.13372 32163658 PMC7292548

[B254] Weckwerth W. Morgenthal K. (2005). Metabolomics: from pattern recognition to biological interpretation. Drug Discov Today 10, 1551–1155. 10.1016/S1359-6446(05)03609-3 16257378

[B255] Wen W. Li D. Li X. Gao Y. Q. Li W. Q. Li H. H.. (2014). Metabolome-based genome-wide association study of maize kernel leads to novel biochemical insights. Nat. Commun. 5:3438. 10.1038/ncomms4438 24633423 PMC3959190

[B256] Williams J. G. K. Kubelik A. R. Livak K. J. Rafalski J. A. Tingey S. V. (1990). DNA polymorphisms amplified by arbitrary primers are useful as genetic markers. Nucleic Acid Res. 18, 6531–6535. 10.1093/nar/18.22.6531 1979162 PMC332606

[B257] Witt S. Galicia L. Lisec J. Cairns J. Tiessen A. Araus J. L.. (2012). Metabolic and phenotypic responses of greenhouse-grown maize hybrids to experimentally controlled drought stress. Mol. Plant 5, 401–417. 10.1093/mp/ssr102 22180467

[B258] Witzel K. Neugart S. Ruppel S. Schreiner M. Wiesner M. Baldermann S. (2015). Recent progress in the use of ‘omics technologies in brassicaceous vegetables. Front. Plant Sci. 6:244. 10.3389/fpls.2015.00244 25926843 PMC4396356

[B259] Wohlbach D. J. Quirino B. F. Sussman M. R. (2008). Analysis of the *Arabidopsis* histidine kinase *ATHK1* reveals a connection between vegetative osmotic stress sensing and seed maturation. Plant Cell 20, 1101–1117. 10.1105/tpc.107.055871 18441212 PMC2390728

[B260] Woolfson M. (2018). The development of structural x-ray crystallography. Phys. Scr. 93, 1–32. 10.1088/1402-4896/aa9c30

[B261] Wu D. Shen Q. Cai S. Chen Z. H. Dai F. Zhang G. (2013). Ionomic responses and correlations between elements and metabolites under salt stress in wild and cultivated barley. Plant Cell Physiol. 54, 1976–1988. 10.1093/pcp/pct134 24058150

[B262] Wu S. Ning F. Zhang Q. Wu X. Wang W. (2017). Enhancing omics research of crop responses to drought under field conditions. Front. Plant. Sci. 8:174. 10.3389/fpls.2017.00174 28261236 PMC5306382

[B263] Xiao J. H. Li J. M. Grandillo S. Ahn S. N. Yuan L. P. Tanksley S. D.. (1998). Identification of trait-improving quantitative trait loci alleles from a wild rice relative, *Oryza rufipogon*. Genetics 150, 899–909. 9755218 10.1093/genetics/150.2.899 PMC1460369

[B264] Xiong L. Schumaker K. S. Zhu J. K. (2002). Cell signaling during cold, drought, and salt stress. Plant Cell 14, 65–S183. 10.1105/tpc.000596 12045276 PMC151254

[B265] Xu X. Liu X. Ge S. Jensen J. D. Hu F. Y. Li X. Y.. (2012). Resequencing 50 accessions of cultivated and wild rice yields markers for identifying agronomically important genes. Nat. Biotechnol. 30, 105–111. 10.1038/nbt.2050 22158310

[B266] Yadav C. B. Pandey G. Muthamilarasan M. Prasad M. (2018). Epigenetics and epigenomics of plants, in Plant Genetics and Molecular Biology. Advances in Biochemical Engineering/Biotechnology, Vol. 64, eds Varshney R. Pandey M. Chitikineni A. (Cham: Springer), 237–261. 10.1007/10_2017_51 29356846

[B267] Yang F. Melo-Braga M. N. Larsen M. R. Jørgensen H. J. L. Palmisano G. (2013). Battle through signaling between wheat and the fungal pathogen *Septoria tritici* revealed by proteomics and phosphoproteomics. Mol. Cell. Proteomics 12, 2497–2508. 10.1074/mcp.M113.027532 23722186 PMC3769326

[B268] Yang L. N. Peng L. Zhang L. M. Zhang L. L. Yang S. S. (2009). A prediction model for population occurrence of paddy stem borer (*Scirpophaga incertulas*), based on Back propagation artificial neural network and principal components analysis. Comput. Electron Agric. 68, 200–206. 10.1016/j.compag.2009.06.003

[B269] Yang W. Guo Z. Huang C. Duan L. Chen G. Jiang N.. (2014). Combining high-throughput phenotyping and genome-wide association studies to reveal natural genetic variation in rice. Nat. Commun. 5:5087. 10.1038/ncomms6087 25295980 PMC4214417

[B270] Yang W. Guo Z. Huang C. Wang K. Jiang N. Fen H.. (2015). Genome wide association study of rice (*Oryza sativa* L.) leaf traits with a high-throughput leaf scorer. J. Exp. Bot. 66, 5605–5615. 10.1093/jxb/erv100 25796084 PMC4585412

[B271] Yang Y. Saand M. A. Abdelaal W. B. Zhang J. Wu Y.i. Li J.. (2020). TRAQ-based comparative proteomic analysis of two coconut varieties reveals aromatic coconut cold-sensitive in response to low temperature. J. proteomics 220:103766. 10.1016/j.jprot.2020.103766 32240811

[B272] Yu J. Holland J. B. McMullen M. D. Buckler E. S. (2008). Genetic design and statistical power of nested association mapping in maize. Genetics 178, 539–551. 10.1534/genetics.107.074245 18202393 PMC2206100

[B273] Zhang C. Yang H. Yang H. (2015). Evolutionary character of alternative splicing in plants. Bioinform. Biol. Insights 9, 47–52. 10.4137/BBI.S33716 26819552 PMC4721685

[B274] Zhang M. Lv D. Ge P. Bian Y. Chen G. Zhu G. (2014a). Phosphoproteome analysis reveals new drought response and defense mechanisms of seedling leaves in bread wheat (*Triticum aestivum* L.). J. Proteomics 109, 290–308. 10.1016/j.jprot.2014.07.010 25065648

[B275] Zhang M. Pinson S. R. Tarpley L. Huang X. Y. Lahner B. Yakubova E.. (2014b). Mapping and validation of quantitative trait loci associated with concentrations of 16 elements in unmilled rice grain. Theor. Appl. Genet. 127, 137–165. 10.1007/s00122-013-2207-5 24231918 PMC4544570

[B276] Zhang X. Huang C. Wu D. Qiao F. Li W. Duan L.. (2017). High throughput phenotyping and QTL mapping reveals the genetic architecture of maize plant growth. Plant Physiol. 173, 1554–1564. 10.1104/pp.16.01516 28153923 PMC5338669

[B277] Zhang X. Zhou S. X. Fu Y. C. Su Z. Wang X. K. Sun C. Q. (2006). Identification of a drought tolerant introgression line derived from Dongxiang common wild rice (*O-rufipogon* Griff.). Plant Mol. Biol. 62, 247–259. 10.1007/s11103-006-9018-x 16845479

[B278] Zhao C. Zhang Y. Du J. Guo X. Wen W. Gu S.. (2019). Crop phenomics: current status and perspectives. Front. Plant Sci. 10:714. 10.3389/fpls.2019.00714 31214228 PMC6557228

[B279] Zhao Q. Feng Q. Lu H. Y. Li Y. Wang A. Tian Q. L.. (2018). Pan-genome analysis highlights the extent of genomic variation in cultivated and wild rice. Nat. Genet. 50, 278–284. 10.1038/s41588-018-0041-z 29335547

[B280] Zhong L. Xu Y. Wang J. (2009). DNA-methylation changes induced by salt stress in wheat *Triticum aestivum*. Afr. J. Biotechnol. 8, 6201–6207. 10.5897/AJB09.1058

[B281] Zhong S. Fei Z. Chen Y. R. Zheng Y. Huang M. Vrebalov J.. (2013). Single-base resolution methylomes of tomato fruit development reveal epigenome modifications associated with ripening. Nat. Biotechnol. 31, 154–159. 10.1038/nbt.2462 23354102

[B282] Zhou Z. Jiang Y. Wang Z. Gou Z. Lyu J. Li W.. (2015). Resequencing 302 wild and cultivated accessions identifies genes related to domestication and improvement in soybean. Nat. Biotechnol. 33, 408–414. 10.1038/nbt.3096 25643055

[B283] Zhu H. G. Cheng W. H. Tian W. G. Li Y. J. Liu F. Xue F.. (2018). iTRAQ-based comparative proteomic analysis provides insights into somatic embryogenesis in *Gossypium hirsutum* L. Plant Mol. Biol. 96, 89–102. 10.1007/s11103-017-0681-x 29214424 PMC5778175

[B284] Zhu Y. N. Shi D. Q. Ruan M. B. Zhang L. L. Meng Z. H. Liu J.. (2013). Transcriptome analysis reveals crosstalk of responsive genes to multiple abiotic stresses in cotton (*Gossypium hirsutum* L.). PLoS ONE 8:e80218. 10.1371/journal.pone.0080218 24224045 PMC3818253

[B285] Ziegler G. Terauchi A. Becker A. Armstrong P. Hudson K. Baxter I. (2013). Ionomic screening of field-grown soybean identifies mutants with altered seed elemental composition. Plant Gen. 6, 1–9. 10.3835/plantgenome2012.07.0012

[B286] Zong W. Zhong X. You J. Xiong L. (2013). Genome-wide profiling of histone H3K4-trimethylation and gene expression in rice under drought stress. Plant Mol. Biol. 81, 175–188. 10.1007/s11103-012-9990-2 23192746

[B287] Zuo W. L. Chao Q. Zhang N. Ye J. R. Tan G. Q. Li B. L.. (2015). A maize wall-associated kinase confers quantitative resistance to head smut. Nat. Genet. 47, 151–157. 10.1038/ng.3170 25531751

